# Influence of Seawater Salts on Magnesium Oxide Hydration: Implications for Carbonation

**DOI:** 10.1021/acsomega.6c00483

**Published:** 2026-09-02

**Authors:** Barbara R. Evans, Dong Youn Chung, Brittany Moseley, Geordan Dorsey, Peng Yang, Andrew G. Stack, Juliane Weber

**Affiliations:** † Chemical Science Division, 6146 Oak Ridge National Laboratory, Oak Ridge, Tennessee 37830, United States; ‡ Roane State Community College, Harriman, Tennessee 37748, United States; § 14589 Fisk University, Nashville, Tennessee 37208, United States

## Abstract

The effects of individual component salts on MgO hydration and subsequent carbonation have not been systematically investigated despite the increase in utilization of seawater and other brines in the production of MgO-containing next-generation cements, fire retardants, and sorbents for CO_2_. Here, we present a study of MgO hydration in the presence of individual saltwater cations at a range of concentrations, as well as binary mixtures at seawater concentrations, and subsequent carbonation. We observed that the presence of the cations increased MgO dissolution rates and that the subsequent carbonation extent of the hydrated material remained constant or even increased. Since the presence of these salts during hydration does not show negative effects on subsequent carbonation in our experimental study, the application of seawater and other brines containing high concentrations of these salts to hydrate MgO in industrial processes is likely feasible.

## Graphical abstract

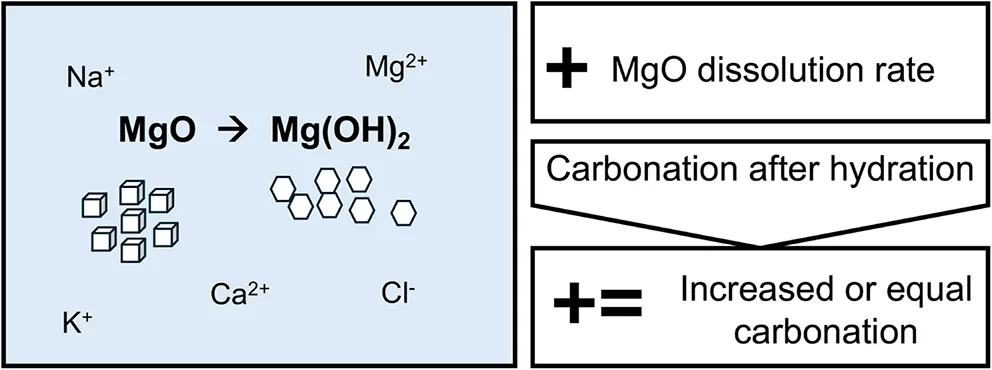

## 1 Introduction

Calcium and magnesium oxides, originating from heating limestone and dolostone, have been used for millennia in the manufacture of cement and concrete due to their significant reactivity, as well as for a variety of other applications. While the most widely used cement is Portland cement based on CaO, MgO-based cements are being investigated with growing interest.^1^ Other uses in the commodity chemical market include magnesium hydroxide, produced by hydration of MgO, which is employed as a fire retardant, while calcium hydroxide is used as a precipitant in chemical processing. Calcium and magnesium oxides have also recently garnered interest as sorbents for the direct air capture of carbon dioxide.^2–4^ The absorbed CO_2_ can be released by heating the carbonated oxides in a looped process, enabling their recycling and recovery of CO_2_.^3–5^

In the utilization of metal oxides for direct air capture, current industrial practice hydrates (meaning reacting the metal oxide with water to form a metal hydroxide) the sorbent prior to carbonation.^5,6^ The use of metal oxides for cements also includes a hydration step, termed slaking.^1^ Using purified water for this purpose is neither cost-effective nor sustainable due to competing water needs. For large-scale applications, it is preferable to use water sources that are not suitable for other purposes. One potential large water resource is seawater, with the main cationic constituents being Na^+^, Mg^2+^, Ca^2+^, and K^+^ (in order of decreasing concentration^7^), and the main anion is chloride. This approach has been recognized as economically advantageous, and using seawater,^35^ desalination reject brine,^8^ and coal mine leachate^9^ for the production of Mg­(OH)_2_ and MgO has been reported; however, these studies have not systematically investigated the effects of the individual component cations. Use of industrial byproducts, such as cement wash water, to increase brine pH can further complicate the composition of the hydration solution.^10^ To optimize the use of seawater and other brines for such purposes, the effects of these elements alone and in combination on MgO hydration and subsequent carbonation reactions must be understood.^11^

There has been extensive research on the effect of aqueous impurities on Ca-based cement slaking^12,13^ but a fundamental understanding of the effect of impurities on the hydration and subsequent carbonation of MgO is missing. For the application of MgO as a sorbent for direct air capture, one study investigated the effect of hydration in the presence of impurities on subsequent carbonation.^14^ Previous studies focused on the effect of aqueous impurities on MgO dissolution rates and Mg­(OH)_2_ formation, for example in the presence of iron,^15^ nickel,^16^ cobalt,^16^ potassium,^17^ sodium,^18^ and calcium.^17^ Most of these studies focused on the application of MgO for contaminant removal from water.^15,19,20^ Other studies focused on how the incorporation of solid impurities or admixtures affects the hydration of MgO.^21,22^ There is also a considerable amount of work on the effects of alkali metal salts on MgO carbonation^23,24^ but these studies were performed at high temperatures (∼300 °C).

What is lacking is knowledge of how the carbonation extent of the hydrated materials is affected by the presence of impurities during hydration. A few prior studies on Fe and seawater have been done. In a previous study, we examined the effects of aqueous iron impurities on MgO hydration and subsequent carbonation.^14^ We found that the presence of dissolved iron leads to the formation of a secondary iron oxide phase, which increases carbonation. A recent study on reactive magnesium oxide cement showed that hydration by seawater does not adversely affect the strength of the resulting cement.^25^ To enable the use of seawater for hydrating MgO during mineral looping for direct air capture or for slaking in cements, it is necessary to determine whether the presence of ions during hydration will have adverse effects on subsequent carbonation. Therefore, in this study, we investigate how impurities common in seawater, such as Ca, Na, K, and Cl, affect the hydration of MgO and its subsequent carbonation.

## 2 Methods

### 2.1 Materials

Water used for experiments and analytical procedures was purified to 18.2 MΩ·cm with a Milli-Q water purification system (Millipore Sigma). MgO single crystals were previously grown at Oak Ridge National Laboratory via a carbon-arc fusion technique.^26^ Powder experiments used commercially available MgO (AlfaAesar, 99.95% purity on a trace-metal basis, primary impurity Ca), referred to as AA MgO below. The as-purchased MgO powder was calcined overnight at 450 °C prior to the experiments to convert any brucite (Mg­(OH)_2_) or Mg-carbonate phases back to MgO. The regenerated powder was then stored under nitrogen atmosphere until the start of the experiments. The calcined MgO powder was reported previously to have an average particle size of 151 ± 41 nm with cubic morphology and a BET surface area of 6.14 m^2^/g.^14^ Solutions of the five major component salts containing CaCl_2_, MgCl_2_, KCl, Na_2_SO_4_, and NaCl—were prepared at the concentrations used for an artificial seawater formulation (see Table S1 for concentrations).^27^ Speciation calculations were performed using PHREEQC^28^ with the llnl database to determine activities and the ionic strength of the solutions. Results of the speciation calculations are given in Table S3.

### 2.2 Single-Crystal Experiments

MgO single crystals were cleaved into small blocks using a razor blade to expose fresh cleavage surfaces. They were then washed with isopropanol under sonication for 5 min to remove mineral dust from cleaving and dried with nitrogen gas. Salt solutions were prepared with deionized water immediately before the start of the experiments. Calcium concentrations ranged from 0.001 to 0.1 M CaCl_2_ (concentration in seawater is ∼0.01 M^7^) and NaCl concentration was 0.1 M (concentration in seawater is ∼0.4 M^7^). Reactors were placed on a shaker table and were incubated at room temperature for 6 to 64 days. At the end of each experiment, the MgO crystal was removed from the solution and washed twice with isopropanol to remove salts and subsequently dried with nitrogen gas. Solutions were filtered using a 0.45 μm pore-size filter and acidified to 2% HNO_3_ for ICP-OES analysis. pH measurements at the end of the experiments were performed with a Denver Instrument 250 Series meter, which was calibrated using a three-point calibration before each measurement. Control experiments without added MgO crystals were conducted in parallel. See Table S4 for an overview of all conducted experiments.

### 2.3 Powder Experiments

MgO powder was added to a beaker while stirring, with either 1 g of powder added to 20 mL of solution or 0.5 g of powder added to 10 mL of solution, to maintain a constant solid-to-liquid ratio of 50 g/L. pH changes were measured during the reaction for 30 min using a Beckman Phi 72 pH meter, calibrated with a two-point calibration. After each experiment, supernatants were decanted and filtered through 0.45 μm-pore-size filters for analysis, while solids were dried in a vacuum oven overnight at 70 °C.

### 2.4 Carbonation Experiments

A custom-built, multivessel system was used to conduct carbonation experiments at 1 bar CO_2_ in the presence of 100% relative humidity. Glass vials containing MgO powder (∼0.2 g) were placed in a Teflon-lined reaction vessel, which was connected to a CO_2_ tank set to deliver CO_2_ gas at 1 bar CO_2_ above atmospheric pressure (gauge). Approximately 2 mL of deionized water was placed at the bottom of the reaction cell, around the glass tubes but not in direct contact with MgO to maintain a constant relative humidity. After the carbonation experiments, samples were dried under vacuum at 70 °C overnight prior to further characterization.

### 2.5 Surface Area Determination by BET

The surface areas of the materials were measured by the Brunauer–Emmett–Teller (BET) nitrogen adsorption method using a Micromeritics instrument interfaced with a SmartVacPrep vacuum drying unit (Micromeritics, Norcross, GA, USA).

### 2.6 TG-MS Analysis

The extent of carbonation in each sample was analyzed by thermogravimetric-mass spectrometry (TGA-MS) using a TA Instruments TGA 5500 coupled with a Discovery MS. Samples from the powder experiments were measured after both hydroxylation and carbonation. The heating rate was 10 °C/min, and samples were heated to 700 °C under flowing argon.

### 2.7 Elemental Analysis with ICP-OES

Metal content of supernatant solutions from powder reactions and crystallization was determined by inductively coupled plasma–optical emission spectroscopy (ICP-OES) using a Thermo Fisher iCAP 7400 ICP-OES spectrometer equipped with a prepFast M5X automatic sample loader with intelligent dilution (Elemental Scientific, Omaha, Nebraska). Trace-metal-grade Ultrex II nitric acid (J.T. Baker brand, Thermo Fisher Scientific) was used to prepare solutions for ICP analysis. Commercial elemental standards for Ca, K, Mg, and Na (High Purity Standards, Charleston, South Carolina, USA) were used to generate seven-point standard curves for each analyte. The experimental solutions were filtered through 0.45-μm-pore-size syringe filters, then diluted 1:50 with 2% HNO_3_ solution for ICP analysis.

### 2.8 Phase Identification via X-ray Diffraction

Powder X-ray diffraction data were collected using a PANalytical XRD with a copper Kα source. The voltage was 45 kV and the current was 40 mA. The scanning time was 10 min, and the 2θ range was 5°–100° or 5°–90°, with a 0.026° step size.

### 2.9 Microscopy

A Zeiss Merlin FE-SEM instrument was used for secondary electron contrast imaging of powder samples at 3 kV. Surface morphology changes of single crystals were identified by using a Keyence VX-3000 profilometer. Atomic force microscopy characterization was performed using an Asylum Research MFP-3D instrument with PNP-TR cantilevers (Nanoworld, resonance frequency of 67 kHz, force constant of 0.32 N/m) in contact mode.

## 3 Results and Discussion

### 3.1 pH Changes during MgO Hydration in the Presence of Seawater Salts

#### 3.1.1 Single Crystals

When MgO comes into contact with water, the solution pH rapidly rises to 10.4. In the presence of dissolved CaCl_2_ in solution during MgO hydration, the solution pH rises to a slightly higher value between 10.5 and 10.7 (Figure 1a). In contrast, when MgO is in contact with 0.1 M NaCl, the final pH is slightly lower at 9.78. Previous studies^29^ have shown that the high pH is due to dissolution of MgO and not equilibration of the protonation state of surface sites. This change in the final equilibrium pH indicates that the presence of salts influences the dissolution mechanism of MgO.

**Figure 1:**
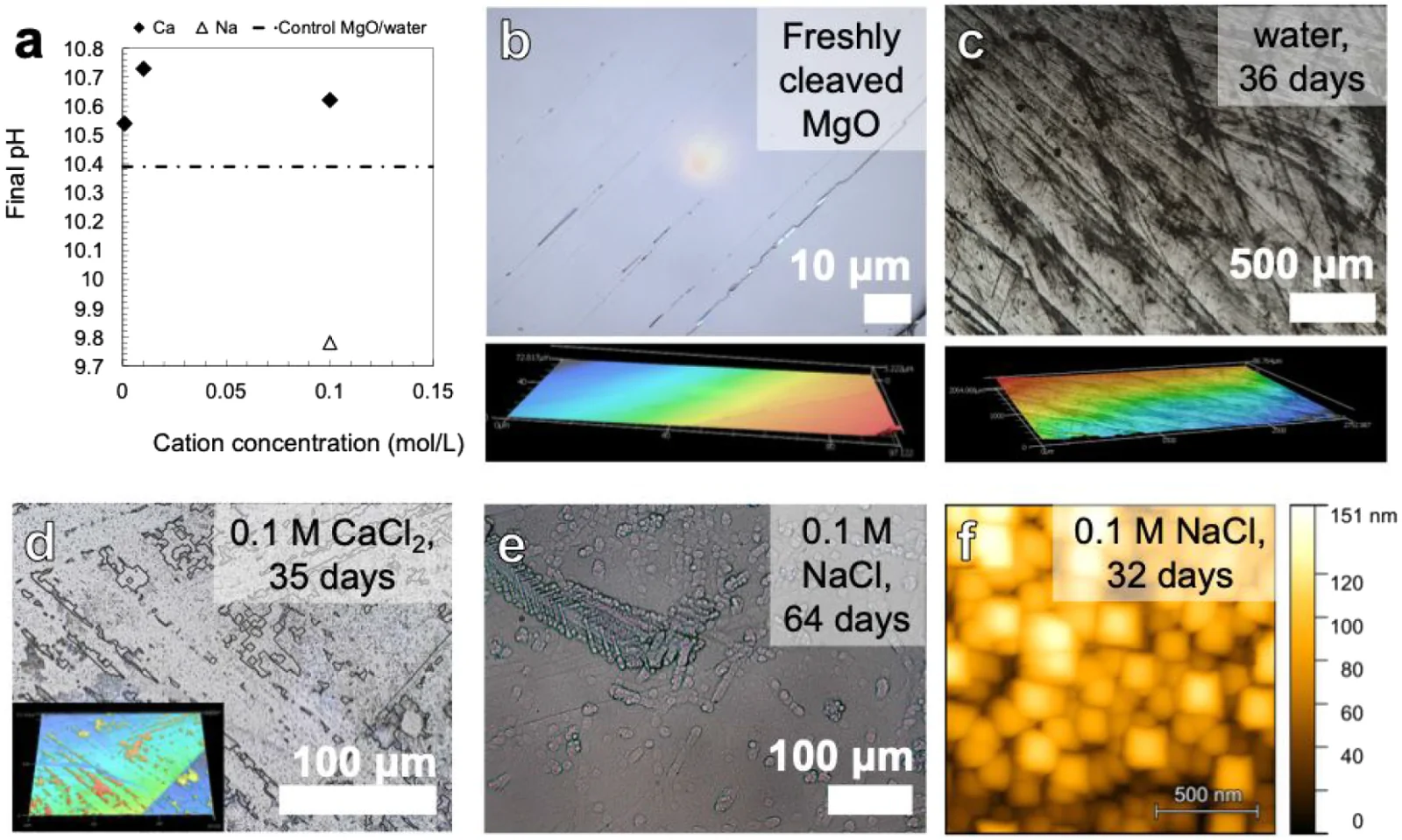
Single-crystal hydration experiments in the presence of salts. (a) Final pH values of MgO crystals reacted with NaCl or CaCl_2_ solutions. The final pH for CaCl_2_ solutions was higher than MgO in contact with deionized water (dotted line), whereas the final pH for NaCl solutions was lower. (b) Freshly cleaved MgO imaged with a profilometer. (c) MgO reacted with deionized water for 36 days. For MgO reacted with 0.1 M CaCl_2_ for 35 days (d), etch pits were visible. Salt precipitates are visible on MgO reacted for 64 days with 0.1 M NaCl (e) and on MgO reacted for 32 days with 0.1 M NaCl, which were imaged using atomic force microscopy (f). The rounded shape of salt precipitates indicates either artifacts during scanning or dissolution of a secondary phase during washing of the crystals with ethanol after the reaction.

#### 3.1.2 Reaction of MgO Powder with Seawater Salts

The pH profiles of MgO powders hydrated in the presence of calcium chloride salts show an initial rapid increase during the first 5 min to 10.5–11.3, followed by a gradual decrease and stabilization during the last 20 min at pH = 10.3–10.8 (Figures S1 and S2). The final pH for pure MgO hydration was observed in previous studies to be ∼10.5–12 depending on the MgO used for the experiment.^14,21,30^ A previous study also found no large effect of stirring speed on final pH,^30^ so it is likely that this reaction is not diffusion-limited. Analysis by the Student’s t test found that the observed variance in the pH values for the CaCl_2_ reactions was not statistically significant between the 0.03 and 0.06 M CaCl_2_ made with anhydrous CaCl_2_ and the other six experiments prepared from the CaCl_2_ hydrate salt (P = 0.074153, higher than the 95% confidence limit of 0.05). These pH changes are similar to our observations on MgO single crystals incubated with CaCl_2_. Both NaCl and KCl show similar effects on the solution pH for concentrations between 0.001 and 0.1 M. When the NaCl concentration was increased to concentrations close to the ones present in seawater, 0.2–0.409 M, the pH increased to 11.0–11.2, then, at 0.485 M NaCl, dropped back to 10.8 (Figure 2b). Binary combinations of NaCl with other sea salts at their seawater concentrations were tested as well (0.409 M NaCl with 0.0103 M CaCl_2_, with 0.0282 M Na_2_SO_4_, and with 0.0523 M MgCl_2_) and caused a greater decrease in final pH compared to 0.409 M NaCl alone, with MgCl_2_ exhibiting the largest effect (pH decrease to 10.1). Analysis of the Mg concentration in solution after the 30 min hydration experiments was used to calculate dissolution rates (Figure 3). Compared to dissolution rates in the absence of salts, MgO dissolution rates are elevated in the presence of salts, which match previous studies on quartz (SiO_2_).^31^ Other studies on feldspars showed that dissolution depends on the nature of the cation in solution.^32^

**Figure 2:**
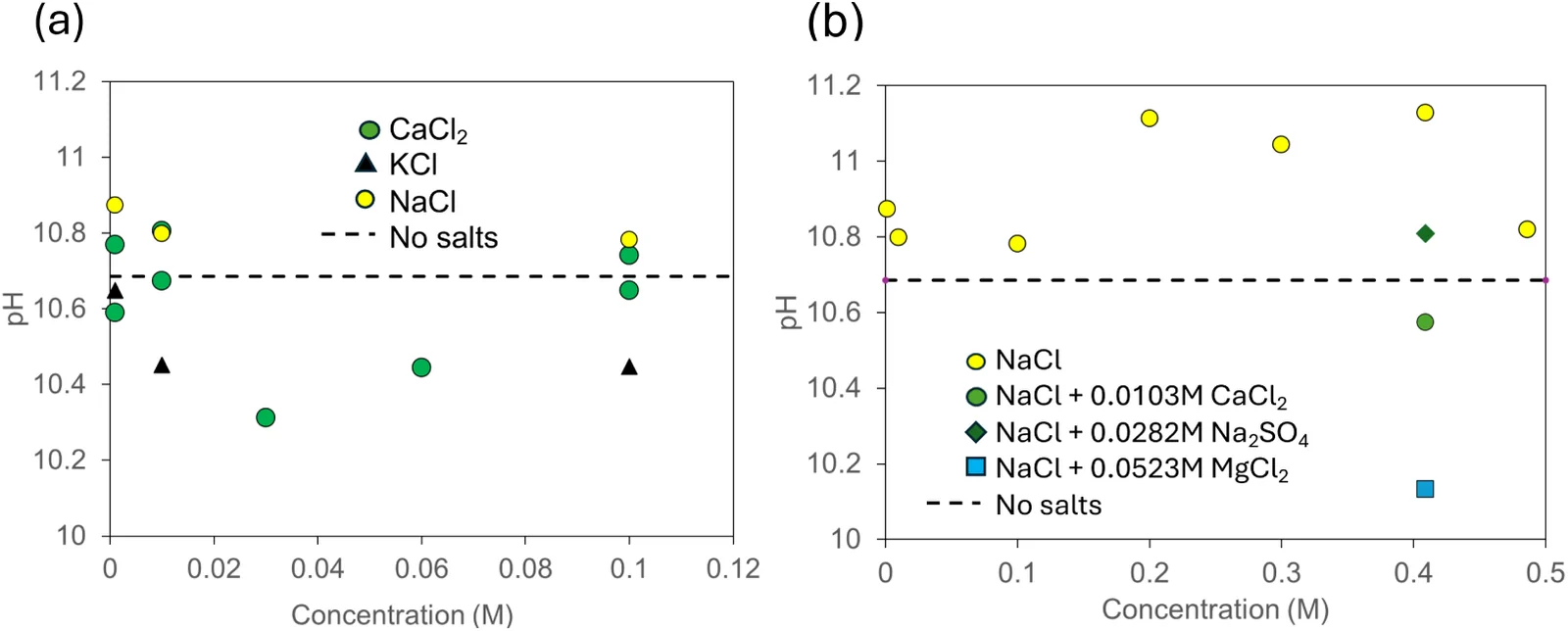
Effect of major sea salts on the averaged pH during the last 20 min of the 30 min hydration reaction of MgO powder. The dotted line denotes the averaged pH during the last 20 min of MgO hydration in deionized water without added salts. (a) The effects of single salts CaCl_2_, KCl, and NaCl at 0.001–0.100 M on the pH of the hydration reactions. (b) The effects of NaCl at 0.001–0.485 M and of two-salt mixtures at their concentrations in seawater: 0.409 M NaCl with 0.0103 M CaCl_2_, 0.409 M NaCl with 0.0282 M Na_2_SO, and 0.409 M NaCl with 0.0523 M MgCl_2_.

**Figure 3:**
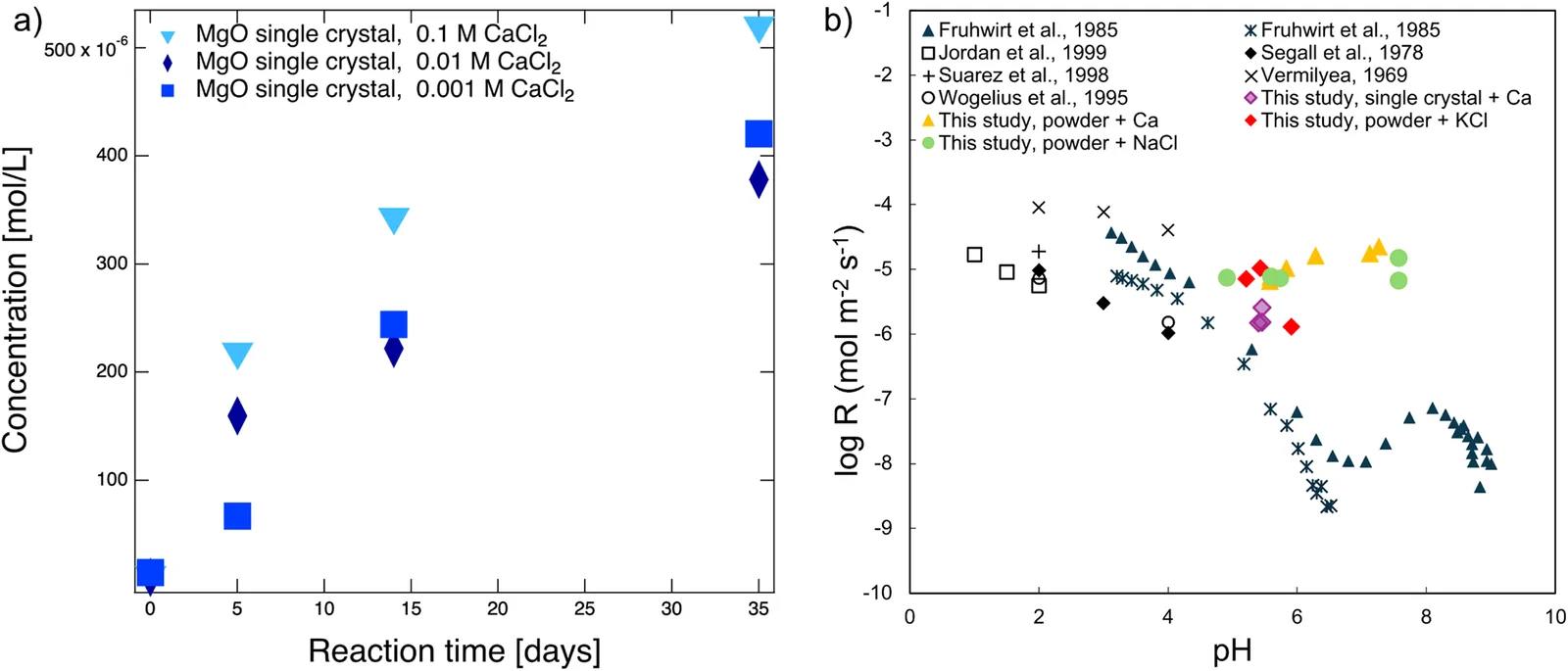
Effect of salt presence on MgO dissolution. (a) Mg concentration as a function of reaction time for single-crystal experiments in the presence of CaCl_2_. (b) Comparison of dissolution rates in the presence of Ca (purple diamonds) for single-crystal MgO and for powders. Figure produced by the authors, adapted from data of MgO dissolution rates in the absence of cations in solution, reviewable in references 33,36–40

### 3.2 Morphology Changes during MgO Single-Crystal Hydration in the Presence of Seawater Salts

Freshly cleaved MgO surfaces showed characteristic features (Figure 1b), as previously reported,^33,34^ including multilayer cleavage steps that are parallel to each other, as dictated by MgO crystallography, and additional steps in zigzag patterns originating from dislocations.^35^ As hydration of MgO crystals in the absence of impurities has been extensively described in a previous publication,^14^ only key results are summarized here. Small features form on the surface, especially at steps (Figure 1c), which can be inferred to be brucite (Mg­(OH)_2_) based on XRD analysis of powder experiments conducted in this study. No dissolution features are visible, indicating that MgO dissolution is passivated by brucite formation.

In contrast, when MgO is hydrated in the presence of CaCl_2_, square etch pits are visible (Figure 1d). ICP-OES analysis (Figure 3) shows the release of Mg into the solution with increasing reaction time, indicating dissolution. The aqueous calcium concentration remains stable (Figure S5), indicating that no secondary Ca phases are formed. The aqueous Mg concentration was fitted to obtain a dissolution rate (Figure S6). The average surface area of MgO single crystals used for the experiments was measured (see SI, Table S6) and dissolution rates in mol·m^–2^ ·s^–1^ were obtained. When comparing our measured dissolution rates with literature data^33,36–40^ (Figure 3b), dissolution rates in the presence of CaCl_2_ are slightly higher than in the absence of salts. At low Ca concentrations (0.001 M CaCl_2_), the dissolution rate can be fit to a first-order rate law, whereas at higher concentrations, a higher-order rate law might be more appropriate. However, we consider the detailed analysis of the effect of aqueous CaCl_2_ on the dissolution kinetics of MgO outside the scope of this study.

In addition to dissolution features, newly formed secondary phases formed after 5 days of reaction in 0.1 M CaCl_2_. These newly formed precipitates were between 3 and 9 μm^2^ in size (Figure 5b). The MgO single crystal, hydrated in the presence of 0.1 M NaCl, showed the formation of surface features (Figure 1e) whose size increased with reaction time. AFM measurements showed the formation of ∼200 nm, rectangular surface features. A previous study at 0.001 M NaCl reported the formation of etch pits within 6 h of reaction.^41^

### 3.3 Morphology Changes during MgO Powder Hydration in the Presence of Seawater Salts

The surface areas of the dried products after hydration of MgO powder in the presence of different seawater salts were determined by BET (Table S6) and compared with the surface area increase after hydration in deionized water, as reported in our previous study.^14^ For MgO hydration in deionized water, we previously observed an increase in surface area from 4.8 to 70.3 m^2^/g previously (1,353%).^14^ Here we used the same starting material (AA MgO powder). The surface area after heating and before hydration was comparable to that of the prior study, at 6 m^2^/g. Therefore, we expect an increase in surface area similar to that observed in our prior study^14^ in the absence of impurities. In the presence of calcium chloride, the surface area changes were about an order of magnitude smaller: 13.9 m^2^/g for 0.001 M CaCl_2_ (+129%); 9.7 m^2^/g for 0.01 M CaCl_2_ (+60%); 7.1 m^2^/g for 0.1 M CaCl_2_ (+17%). In the presence of KCl, surface area changes were about half those observed for hydration with pure water: 39.5 m^2^/g for 0.001 M KCl (+553%); 20.5 m^2^/g for 0.01 M KCl (+239%); and 10.6 m^2^/g for 0.1 M KCl (+75%), showing a clearly concentration-dependent effect: with increasing KCl concentration, there was a smaller increase in surface area. For sodium chloride, surface area changes were more than an order of magnitude smaller: 9.0 m^2^/g for 0.001 M NaCl (+49%); 8.7 m^2^/g for 0.01 M NaCl (+43%); 10.0 m^2^/g for 0.1 M NaCl (+65%), and 11.8 m^2^/g for 0.409 M NaCl (+95.5%). Thus, for NaCl electrolytes, no clear concentration-dependent effect on surface area was observed.

These differences between samples hydrated with NaCl and with KCl can be correlated with the surface morphology observed by SEM (Figures 4 and 5). SEM imaging of products from hydration experiments in the presence of KCl showed the formation of thin platelets (Figure 4c), which were identified by XRD as Mg­(OH)_2_ (Figure 4a). Consistent with a previous study on the effect of seawater on MgO hydration,^42^ the newly formed hydrated phases appear thinner and more bent in the presence of salts than those formed during MgO hydration in deionized water.

**Figure 4:**
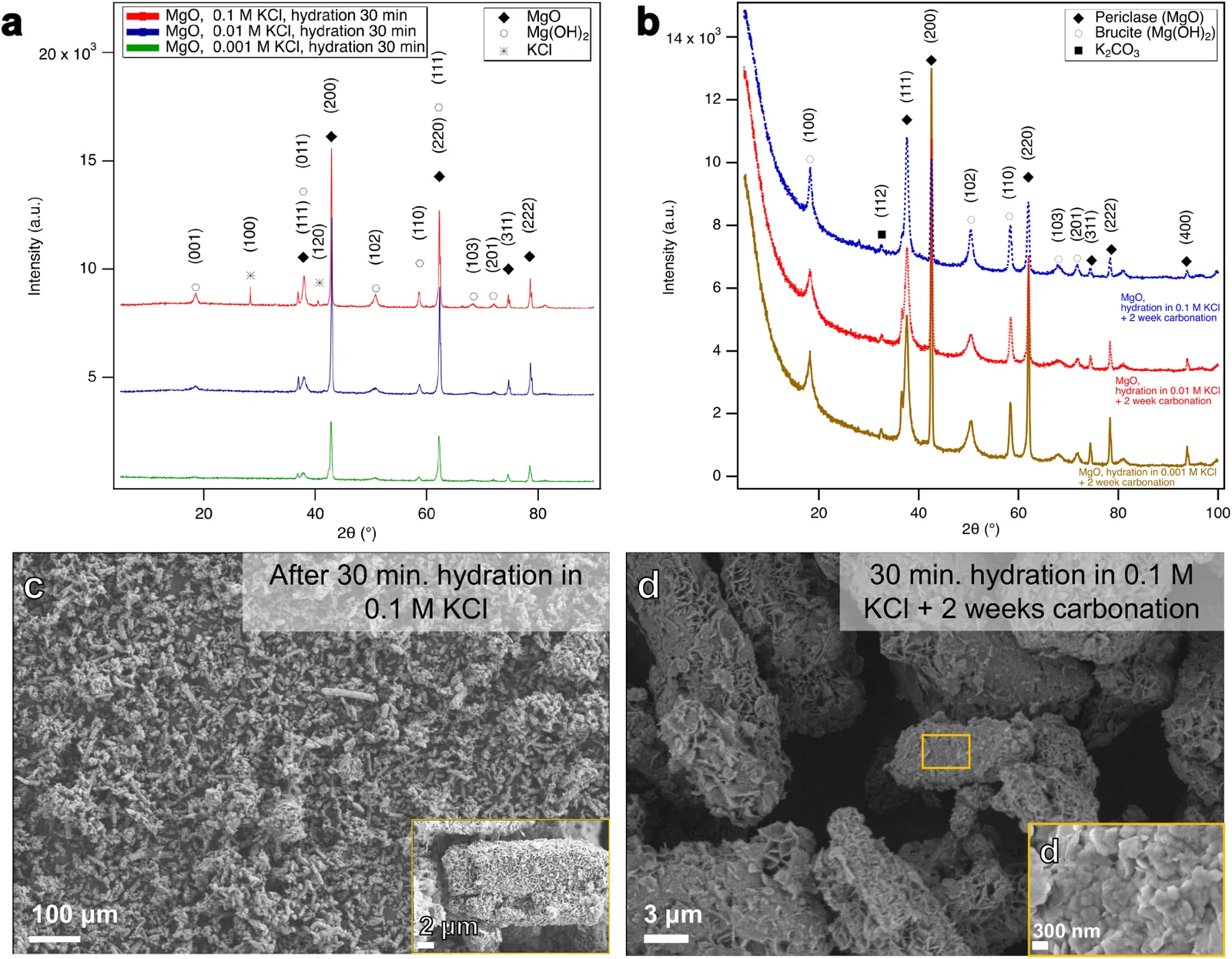
XRD and SEM results showing the effect of KCl on MgO hydration and subsequent carbonation. (a) XRD spectra of MgO hydrated in the presence of KCl showing the presence of Mg­(OH)_2_ for all concentrations. At higher KCl concentrations, KCl (sylvite) peaks were observed as well. (b) XRD characterization after carbonation showed a small amount of K_2_CO_3_ in addition to brucite and MgO peaks. (c) Scanning electron microscopy images showing typical Mg­(OH)_2_ platelet morphology. (d) SEM images of carbonated MgO after hydration in 0.1 M KCl. Higher magnification images (d inset) show coating of particles.

**Figure 5:**
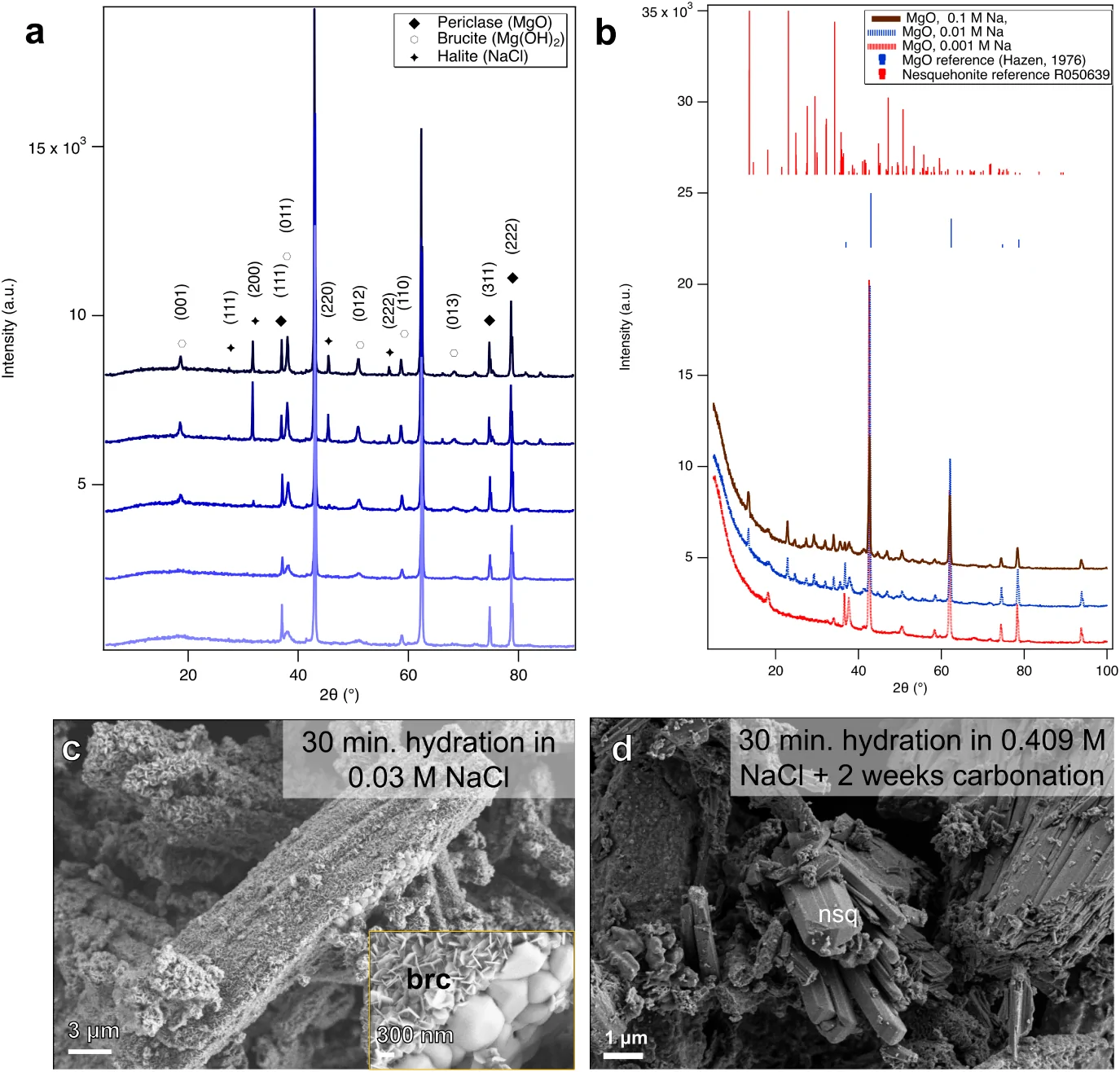
XRD and SEM results showing the effect of NaCl on MgO hydration and subsequent carbonation. (a) XRD characterization of MgO hydrated in the presence of NaCl showing both brucite (Mg­(OH)_2_) and halite (NaCl) formation. Aqueous NaCl concentration is increasing from bottom to top. (b) XRD characterization shows that the main carbonation product is nesquehonite. (c) Scanning electron microscopy characterization of MgO hydrated in the presence of NaCl. Both typical Mg­(OH)_2_ (brucite, brc) platelet morphology and large halite crystals are visible. (d) SEM images of carbonated MgO after hydration in 0.409 M NaCl. Typical nesquehonite (nsq) morphology is visible.

The products from the hydration of MgO powder in the presence of NaCl showed the typical brucite platelet morphology (Figure 5), but with additional dispersed larger crystals (∼400 nm) with differing morphology. XRD analysis showed the presence of halite (NaCl) (Figure 5a) in the sample hydrated in the presence of NaCl. The dispersed larger crystals were beam-sensitive under the SEM electron beam, which is typical of alkali halides.^43^ These dispersed larger crystals could therefore be halite, but local chemical analysis would be needed to verify this. MgO hydrated in the presence of calcium chloride showed typical brucite platelet morphology (Figure S26). No secondary phases were detected.

### 3.4 Effect of Seawater Salts on MgO Carbonation Efficiency

To test the potential effects of cations present in seawater during hydration on the extent of subsequent carbonation of MgO, carbonation experiments were conducted. Mass loss by TGA for MgO hydrated in deionized water was previously reported to be 23%.^14^ For most salts, increased carbonation was observed after hydration in the presence of salts. To compare the different salts directly, we calculated the relative change in mass loss compared to the mass loss for carbonation after hydration in pure water (Δm/m_0_) by using this formula: (% mass loss in the presence of salt – % mass loss in the absence of salt)/% mass loss in the absence of salt (Table 1). Substantial increases in carbonation of 31% and 42% were obtained for MgO hydrated in the presence of 0.1 and 0.409 M NaCl, respectively. The increase in carbonation was lower for binary salt combinations with CaCl_2_, MgCl_2_, and Na_2_SO_4_ at their seawater concentrations in 0.409 M NaCl than that obtained for NaCl alone. The only decrease was observed for carbonation in the presence of 0.001 M KCl, with the higher concentrations of 0.01 and 0.1 M giving the lowest increases among the salts tested. This observation contrasts with the decrease in surface area of the MgO hydroxylation products with increasing KCl concentration. In summary, the presence of the tested seawater salts during hydration of MgO was found to generally increase subsequent carbonation, with NaCl at 0.1 M and at its seawater concentration showing the highest increase, while KCl gave the lowest increase among the salts tested.

**Table 1:** Effect of the Presence of Seawater Salts during MgO Hydration on Subsequent Carbonation Efficiency^a^

Salt	Concentration (M)	Mass Loss (%)	Δm/m_0_
None^b^	N/A	23	N/A
CaCl_2_	0.001	25	7
CaCl_2_	0.01	27	14
CaCl_2_	0.1	24	4
KCl	0.001	22	–5
KCl	0.01	24	4
KCl	0.1	26	12
NaCl	0.001	24	3
NaCl	0.01	25	8
NaCl	0.1	31	34
NaCl	0.409	42	81
NaCl, CaCl_2_	0.409, 0.0103	33	41
NaCl, MgCl_2_	0.409, 0.0532	37	59
NaCl, Na_2_SO_4_	0.409, 0.0283	27	17

a The table gives the salt used during hydration of MgO, its concentration in M, the mass loss (%) measured by TGA-MS, and the relative change in mass loss compared with the mass loss for carbonation after hydration in pure water (calculated by this formula: (% mass loss in the presence of salt – % mass loss in the absence of salt)/% mass loss in the absence of salt).

b Deionized purified (18.2 MΩ·cm) water without salts, data presented in a previous study.^11^

### 3.5 Effect of Seawater Salts on MgO Carbonation Products

XRD analysis of the carbonation products of MgO hydrated in the presence of CaCl_2_ showed mainly nesquehonite, but brucite and portlandite (Ca­(OH)_2_) were also present (Figure 6). SEM imaging showed that the original platelets had started to dissolve and that a surface coating had formed. For carbonation of MgO hydrated in the presence of NaCl, the main carbonation product was also nesquehonite (MgCO_3_·3H_2_O) (Figure 5). Samples hydrated in KCl showed formation of K_2_CO_3_ in XRD measurements and surface coatings in SEM imaging (Figure 4).

**Figure 6:**
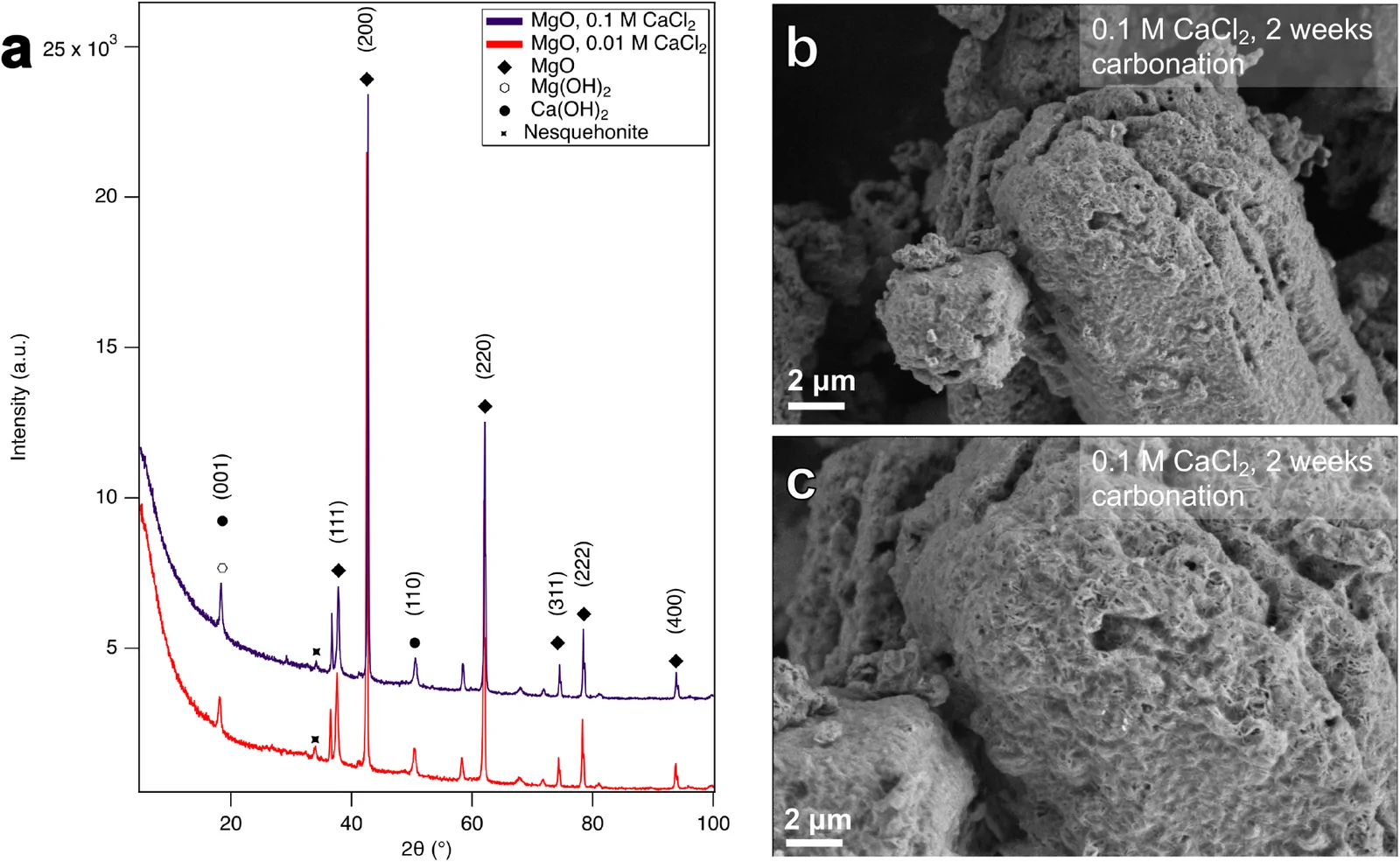
Characterization of carbonation products for MgO hydrated in the presence of CaCl_2_, carbonated for 2 weeks at 100% RH and 1 bar CO_2_. (a) XRD characterization shows some nesquehonite formation. (b) and (c) SEM images of carbonated MgO after hydration in 0.1 M CaCl_2_.

## 4 Discussion

Our results show that the presence of aqueous cations during the hydration of MgO affects both the hydration reaction and subsequent carbonation. First, we tested whether the ionic strength of the solution controls the final pH, the surface area after hydration, the carbonation extent, or the dissolution rate of MgO (Figure S27), but no clear correlation was found, indicating that the nature of the salt might be more important than the ionic strength. Next, to identify whether carbonation is affected by changes in the surface area, we compared the changes in the surface area and carbonation extent. We find that both are highly dependent on the individual salt present (Figure 7). A correlation between an increase in carbonation extent and an increase in surface area was only observed for NaCl as the concentration increased from 0.001 to 0.409 M; however, the surface area of the hydrated reaction product did not change significantly. As KCl concentrations decreased from 0.1 to 0.001 M, an increase in surface area was observed, but carbonation decreased with increasing surface area. For calcium chloride, neither surface area nor carbonation extent increased as the CaCl_2_ concentration was increased. One potential reason could be that the increased dissolution of MgO in the presence of CaCl_2_ (observed for single crystals) changes the Mg­(OH)_2_ formation rate, which leads to changes in the surface area of the hydrated material. A previous study on the effects of salts (Na and K at concentrations of up to 2.4 mol/L) on the recrystallization of Mg­(OH)_2_ showed that the presence of salts leads to a decrease in particle size and a decrease in surface area due to a change in morphology toward hexagonal flakes.^44^

**Figure 7:**
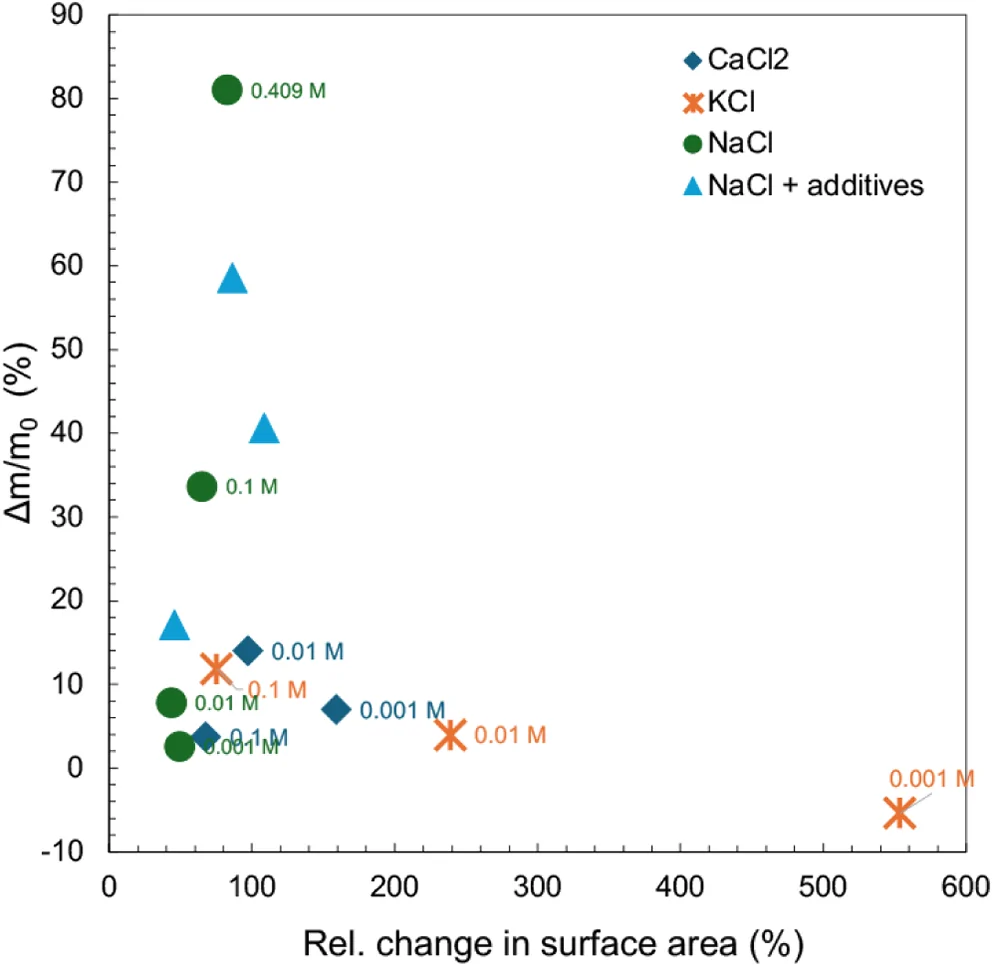
Correlation of percent change in surface area by hydration of MgO in the presence of salts to percent change in carbonation measured as percent weight loss .

## 5 Conclusions

Our investigations of the effects of the major component cations of seawater on MgO hydration and subsequent carbonation have found that their presence can increase carbonation efficiency in laboratory-scale experiments, which may be contrary to conventional wisdom. These results have important implications for the use of seawater and other brines in processes that utilize MgO hydration and carbonation, considering that additional factors, such as the thickness of the sorbent layer, biofilm growth, and other variables, will influence practical and industrial applications.

The use of seawater and similar brines for hydration of MgO before carbonation has the potential to be a cost-effective pretreatment, thus potentially improving the sustainability of direct air capture via mineral looping. The observed changes in dissolution, crystallization, and pH for the different cations also provide new insights into these dynamic reactions that are relevant to low-concentration CO_2_ reactions, the performance of cement formulations, and the production of Mg­(OH)_2_ fire retardants. Our lab-scale investigation of the individual component salts at varying concentrations extends the relevance of these results to the evaluation of industrial byproducts and water sources with concentrations and compositions that differ from seawater.

## Supplementary Material



## Data Availability

Evans, Barbara; Chung, Dong Youn; Moseley, Brittany; Yang, Peng; Dorsey, Geordan; Stack, Andrew; Weber, Juliane (2025), “Data associated with manuscript’Influence of Seawater Salts on Magnesium Oxide Hydration: Implications for Carbonation’”, Mendeley Data, v1, DOI: 10.17632/khhtfnf67t.1. Additional data are available from the corresponding author upon reasonable request.

## Notes

Overview of simulated seawater composition; overview of single-crystal and powder hydration experiments; pH monitoring results; additional information on the ICP-OES results; and plots of TGA-MS data (PDF)

## ∥

NASA Johnson Space Center, Houston, 77058, TX, USA

## ⊥

Brookhaven National Laboratory, Upton, 11973, NY, USA

## References

[1] Walling S. A., Provis J. L. (2016). Magnesia-based cements: a journey of 150 years, and
cements for the future?. Chem. Rev..

[2] McQueen N., Kelemen P., Dipple G., Renforth P., Wilcox J. (2020). Ambient weathering
of magnesium oxide for CO_2_ removal from air. Nat. Commun..

[3] Dostie L., Rausis K., Power I. M. (2024). Passive
direct air capture using
calcium oxide powder: The importance of water vapor. J. Clean. Prod..

[4] Rausis K., Stubbs A. R., Power I. M., Paulo C. (2022). Rates of atmospheric
CO_2_ capture using magnesium oxide powder. Int. J. Greenhouse Gas Control.

[5] McQueen N., Drennan D. (2024). The use of warehouse automation technology
for scalable
and low-cost direct air capture. Front. Clim..

[6] Paulsen M. M., Nielsen S. G. R., Tilsted F. J., Pedersen T. H. (2025). Is solid
calcium
looping a scalable technology for mega-ton carbon dioxide removal?. J. CO2 Util..

[7] Nordstrom, D. K. ; Plummer, L. N. ; Wigley, T. M. L. ; Wolery, T. J. ; Ball, J. W. ; Jenne, E. A. ; Bassett, R. L. ; Crerar, D. A. ; Florence, T. M. ; Fritz, B. A Comparison of Computerized Chemical Models for Equilibrium Calculations in Aqueous Systems. In Chemical Modeling in Aqueous Systems: Speciation, Sorption, Solubility, and Kinetics; American Chemical Society, 1979.

[8] Dong H., Unluer C., Yang E.-H., Al-Tabbaa A. (2017). Synthesis
of reactive MgO from reject brine via the addition of NH_4_OH. Hydrometallurgy.

[9] Turek M., Gnot W. (1995). Precipitation of magnesium
hydroxide from brine. Ind. Eng. Chem. Res..

[10] Du Z., Yang E.-H., Unluer C. (2024). Investigation of the properties of
Mg (OH)_2_ extracted from magnesium-rich brine via the use
of an industrial by-product. Cem. Concr. Compos..

[11] Bhojwani S., Topolski K., Mukherjee R., Sengupta D., El-Halwagi M. M. (2019). Technology
review and data analysis for cost assessment of water treatment systems. Sci. Total Environ..

[12] Gallala W., Gaied M., Tlili A., Montacer M. (2008). Factors influencing
the reactivity of quicklime. Proc. Inst. Civ.
Eng.: Constr. Mater..

[13] Shang X., Ye G., Zhai P., Wang Q., Zhang C. (2016). Hydration behavior
of calcium alumina cements with different amounts of impurities. Interceram Int. Ceram. Rev..

[14] Weber J., Moseley B., Yuan K., Evans B. R., Starchenko V., Rodriguez E. T., Chung D. Y., Boebinger M. G., McGuire M. A., Yumnam G. (2025). Influence of Dissolved
Iron in Solution on MgO Hydroxylation and Carbonation. J. Phys. Chem. C.

[15] O’Connor J. T., Benson B. E. (1970). Iron removal using magnesium oxide. J. Sani. Eng. Div..

[16] de
Repentigny C., Courcelles B., Zagury G. J. (2018). Spent MgO-carbon
refractory bricks as a material for permeable reactive barriers to
treat a nickel-and cobalt-contaminated groundwater. Environ. Sci. Pollut. Res..

[17] Amaral L., Oliveira I., Salomão R., Frollini E., Pandolfelli V. (2010). Temperature
and common-ion effect on magnesium oxide (MgO) hydration. Ceram. Int..

[18] Li Y., Zhang G., Hou D., Wang Z. (2021). Nanoscale insight on
the initial hydration mechanism of magnesium phosphate cement. Constr. Build. Mater..

[19] Schiller J. E., Tallman D. N., Khalafalla S. E. (1984). Mineral processing water treatment
using magnesium oxide. Environ. Prog..

[20] Szymoniak L., Claveau-Mallet D., Haddad M., Barbeau B. (2022). Application of magnesium
oxide media for remineralization and removal of divalent metals in
drinking water treatment: A review. Water.

[21] Pettauer M., Baldermann A., Eder S., Dietzel M. (2024). Hydration of MgO: reaction
kinetics and pH control on brucite crystal morphology. Cryst. Growth Des..

[22] Kuhn R., Lothenbach B., Lura P., Habert G., Bernard E. (2026). Effects of
carbonate and phosphate admixtures on MgO dissolution and Mg (OH)
2 precipitation. Cem. Concr. Res..

[23] Harada T., Simeon F., Hamad E. Z., Hatton T. A. (2015). Alkali metal nitrate-promoted
high-capacity MgO adsorbents for regenerable CO_2_ capture
at moderate temperatures. Chem. Mater..

[24] Santos H.
S., Nguyen H., Bojdys M. J., Esmaeili P., Sirviö J. A., Kinnunen P. (2025). Insights into the mechanism of nitrate salt-mediated
MgCO_3_ formation. Phys. Chem. Chem.
Phys..

[25] Wang X., Krishnan P., Varghese S., Singh I., Chinyanga E., Celik K. (2026). Impact of seawater
on the hydration and carbonation of reactive magnesium
oxide cement. Cem. Concr. Res..

[26] Abraham M., Butler C., Chen Y. (1971). Growth of
High-Purity and Doped Alkaline
Earth Oxides: I. MgO and CaO. J. Chem. Phys..

[27] Kester D.
R., Duedall I. W., Connors D. N., Pytkowicz R. M. (1967). Preparation
of artificial seawater. Limnol. Oceanogr..

[28] Parkhurst, D. L. ; Appelo, C. A. J. User’s guide to PHREEQC (Version 2): a computer program for speciation, batch-reaction, one-dimensional transport, and inverse geochemical calculations; U.S. Geological Survey, 1999.

[29] Adapa S., Yuan K., Evans B. R., Weber J., Irle S., Anovitz L. M., Stack A. G. (2025). Acidities
of MgO surface sites: implications
for the formation mechanism of Mg (OH)_2_. J. Mater. Chem. A.

[30] Cavrot R., Diouf A., Baumier C., Goniakowski J., Giordano L., Stankic S. (2026). Shape Evolution of
MgO Nanocubes
via Rapid pH Self-Adjustment at Oxide–Water Interfaces. ACS Nanosci. Au.

[31] Dove P. M. (1999). The dissolution
kinetics of quartz in aqueous mixed cation solutions. Geochim. Cosmochim. Acta.

[32] Bagheri M., Lothenbach B., Shakoorioskooie M., Scrivener K. (2022). Effect of
different ions on dissolution rates of silica and feldspars at high
pH. Cem. Concr. Res..

[33] Jordan G., Higgins S. R., Eggleston C. M. (1999). Dissolution
of the periclase (001)
surface; a scanning force microscope study. Am. Mineral..

[34] Abriou D., Creuzet F., Jupille J. (1996). Characterization of cleaved MgO (100)
surfaces. Surf. Sci..

[35] Robins J., Rhodin T., Gerlach R. (1966). Dislocation
structures in cleaved
magnesium oxide. J. Appl. Phys..

[36] Vermilyea D. A. (1969). The dissolution
of MgO and Mg (OH)_2_ in aqueous solutions. J. Electrochem. Soc..

[37] Suárez M. F., Compton R. G. (1998). Dissolution of magnesium oxide in aqueous acid: an
atomic force microscopy study. J. Phys. Chem.
B.

[38] Wogelius R. A., Refson K., Fraser D. G., Grime G. W., Goff J. P. (1995). Periclase
surface hydroxylation during dissolution. Geochim.
Cosmochim. Acta.

[39] Fruhwirth O., Herzog G., Hollerer I., Rachetti A. (1985). Dissolution and hydration
kinetics of MgO. Surf. Technol..

[40] Fedoročková A., Raschman P. (2008). Effects of
pH and acid anions on the dissolution kinetics
of MgO. Chem. Eng. J..

[41] Giner I., Ozcan O., Grundmeier G. (2014). In situ AFM
studies of the stability
of MgO (1 0 0) in aqueous electrolytes. Corros.
Sci..

[42] Tang X., Guo L., Liu Q., Li Y., Li T., Zhu Y. (2015). Morphology
analysis of magnesium hydroxide prepared by magnesium oxide hydration
within seawater. Cryst. Res. Technol..

[43] Egerton R. F., Li P., Malac M. (2004). Radiation damage in the TEM and SEM. Micron.

[44] Weng C., Song X., Zhu H., Luo X. (2024). Influence
of alkali
metal ions (K^+^ and Na^+^) on the preparation of
magnesium hydroxide hexagonal flakes. RSC Adv..

