# Supplementary material for: Influence of Seawater Salts on Magnesium Oxide Hydration: Implications for Carbonation
Source: ACS Omega. 2026 Sep 2;11(36):53760–8. doi: 10.1021/acsomega.6c00483 (PMC13584149; doi:10.1021/acsomega.6c00483)
Supplement: Supplementary file 1 [file ao6c00483_si_001.pdf]

Supporting Information

for

# Influence of Seawater Salts on Magnesium Oxide Hydration: Implications for Carbonation

Barbara R. Evans[1], Dong Youn Chung[1], Brittany Moseley[2], Geordan Dorsey[3], Peng Yang[1#], Andrew G. Stack[1], Juliane Weber[1*]

[†]Chemical Science Division, Oak Ridge National Laboratory, Oak Ridge, USA.
[2]Roane State Community College, Harriman, TN, 37748, USA.
[3]Fisk University, Nashville, TN, USA.

**Table of Contents**

1. Additional Material & Methods ....................3

2. Composition of Simulated Seawater ....................3

3. Overview of Conducted Single Crystal Hydration Experiments ....................5

4. MgO Powder Hydration Experiments ....................6

5. pH Monitoring Results ....................7

6. Additional ICP-OES Results ....................9

7. Calculation of Dissolution Rate ....................10

8. Additional Microscopy Results ....................11

9. TGA-MS Results ....................14

10. Surface Area Measurements for Powder Experiments ....................22

11. ICP-OES Analysis of MgO Hydration Supernatants ....................23

12. Additional SEM Characterization ....................26

13. References ....................27

## 1. Additional Material & Methods

The purities of the salts as given by the manufacturers were: $CaCl_2$ anhydrous 99.995% and $CaCl_2$•(4-6) $H_2O$ (Puratronic, Alfa Aesar); $MgCl_2$ hexahydrate 98.0-101.0 % (USP, J. T. Baker); KCl 99-100.5% (Sigma-Aldrich); $Na_2SO_4$ 99.5%, (ACS, Fisher); NaCl >99.9% (Fisher Biotech).

## 2. Composition of Simulated Seawater

Table S 1 The six major components of seawater and their concentrations.[1]

| Component | Molarity | Formal weight (g/mol) | g/L | ppm |
|---|---|---|---|---|
| Na | 0.468 | 22.98977 | 10.75921 | 10759.21 |
| K | 0.009897 | 39.102 | 0.386992 | 386.9925 |
| Cl | 0.545878 | 35.453 | 19.353 | 19353 |
| Mg | 0.05323 | 24.305 | 1.293755 | 1293.755 |
| Ca | 0.0103 | 40.08 | 0.412824 | 412.824 |
| Sulfate | 0.02823 | 96.0576 | 2.711706 | 2711.706 |

Table S 2 Concentration of the five major salts in the artificial seawater formulation used in this work.[1]

| Salt | Concentration (M) |
|---|---|
| NaCl | 0.409 |
| KCl | 0.009897 |
| $MgCl_2$ | 0.05323 |
| $Na_2SO_4$ | 0.0282 |
| $CaCl_2$ | 0.0103 |

Table S 3 Speciation calculation results showing main cation concentrations (brackets), activities (braces) and ionic strength for solutions used in hydration experiments.

| Experiment name | $[Na^+]$ | $\{Na^+\}$ | $[Cl^-]$ | $\{Cl^-\}$ | $[Ca^{2+}]$ | $\{Ca^{2+}\}$ | $[Mg^{2+}]$ | $\{Mg^{2+}\}$ | $[K^+]$ | $\{K^+\}$ | Ionic strength calculated using PHREEQC (mol/kgw) |
|---|---|---|---|---|---|---|---|---|---|---|---|
| 0.001 M NaCl | 1.00E-03 | 9.65E-04 | 1.00E-03 | 9.65E-04 | | | | | | | 0.0010 |
| 0.01 M NaCl | 9.99E-03 | 9.01E-03 | 9.99E-03 | 8.99E-03 | | | | | | | 0.0100 |
| 0.1 M NaCl | 9.96E-02 | 7.73E-02 | 9.96E-02 | 7.57E-02 | | | | | | | 0.0996 |
| 0.2 M NaCl | 1.99E-01 | 1.46E-01 | 1.99E-01 | 1.41E-01 | | | | | | | 0.1988 |
| 0.3 M NaCl | 2.98E-01 | 2.11E-01 | 2.98E-01 | 2.02E-01 | | | | | | | 0.2979 |
| 0.409 M NaCl | 4.06E-01 | 2.80E-01 | 4.06E-01 | 2.66E-01 | | | | | | | 0.4059 |
| 0.486 M NaCl | 4.82E-01 | 3.29E-01 | 4.82E-01 | 3.11E-01 | | | | | | | 0.4822 |
| 0.001M $CaCl_2$ | | | 2.00E-03 | 1.88E-03 | 1.00E-03 | 7.92E-04 | | | | | 0.0030 |
| 0.01 M $CaCl_2$ | | | 2.00E-02 | 1.69E-02 | 9.99E-03 | 5.45E-03 | | | | | 0.0300 |
| 0.03 M $CaCl_2$ | | | 6.00E-02 | 4.61E-02 | 2.99E-02 | 1.24E-02 | | | | | 0.0900 |
| 0.06 M $CaCl_2$ | | | 1.20E-01 | 8.60E-02 | 5.99E-02 | 2.05E-02 | | | | | 0.1801 |
| 0.1 M $CaCl_2$ | | | 2.01E-01 | 1.36E-01 | 9.98E-02 | 2.97E-02 | | | | | 0.3005 |
| 0.001 M KCl | | | 1.00E-03 | 9.65E-04 | | | | | 1.00E-03 | 9.65E-04 | 0.0010 |
| 0.01M KCl | | | 1.00E-02 | 9.00E-03 | | | | | 1.00E-02 | 9.00E-03 | 0.0100 |
| 0.1 M KCl | | | 1.01E-01 | 7.64E-02 | | | | | 1.01E-01 | 7.64E-02 | 0.1006 |
| 0.001 M $CaCl_2$ | | | 2.00E-03 | 1.88E-03 | 1.00E-03 | 7.92E-04 | | | | | 0.0030 |
| 0.01 M $CaCl_2$ | | | 2.00E-02 | 1.69E-02 | 9.99E-03 | 5.45E-03 | | | | | 0.0300 |
| 0.100 M $CaCl_2$ | | | 2.01E-01 | 1.36E-01 | 9.98E-02 | 2.97E-02 | | | | | 0.3005 |
| 0.409 M NaCl | 4.06E-01 | 2.80E-01 | 4.06E-01 | 2.66E-01 | | | | | | | 0.4059 |
| 0.409 M NaCl + 0.0103 M $CaCl_2$ | 2.06E-01 | 1.49E-01 | 2.16E-01 | 1.51E-01 | 5.07E-03 | 1.64E+00 | | | | | 0.2208 |
| 0.409 M NaCl + 0.0282 M $Na_2SO_4$ | 2.30E-01 | 1.66E-01 | 2.05E-01 | 1.42E-01 | | | | | | | 0.2400 |
| 0.409 M NaCl + 0.0523 M $MgCl_2$ | 2.05E-01 | 1.46E-01 | 2.56E-01 | 1.74E-01 | | | 2.46E-02 | 8.90E-03 | | | 0.2805 |

## 3. Overview of Conducted Single Crystal Hydration Experiments

Table S 4 Overview of conducted single crystal hydration experiments.

| Experiment name | Solution composition | Duration (days) |
|---|---|---|
| MgO_NaCl_5d | 0.1 M NaCl | 5 |
| MgO_NaCl_15d | | 15 |
| MgO_NaCl_36d | | 36 |
| MgO_NaCl_64d | | 64 |
| MgO_0p1Ca_5d | 0.1 M $CaCl_2$ | 5 |
| MgO_0p1Ca_14d | | 14 |
| MgO_0p1Ca_36d | | 36 |
| MgO_0p1Ca_64d | | 64 |
| MgO_0p01Ca_5d | 0.01 M $CaCl_2$ | 5 |
| MgO_0p01Ca_14d | | 14 |
| MgO_0p01Ca_36d | | 36 |
| MgO_0p01Ca_64d | | 64 |
| MgO_0p001Ca_5d | 0.001 M $CaCl_2$ | 5 |
| MgO_0p001Ca_14d | | 14 |
| MgO_0p001Ca_36d | | 36 |
| MgO_0p001Ca_64d | | 64 |
| Control_0p1NaCl | 0.1 M NaCl – no MgO | Sampled at 5,14, 36 and 64 |
| Control_0p1Ca | 0.1 M $CaCl_2$ – no MgO | Sampled at 5,14, 36 and 64 |
| Control_0p01Ca | 0.01 M $CaCl_2$ – no MgO | Sampled at 5,14, 36 and 64 |
| Control_0p001Ca | 0.001 M $CaCl_2$ – no MgO | Sampled at 5,14, 36 and 64 |

## 4. MgO Powder Hydration Experiments

Table S 5 Overview of conducted powder hydration experiments.

| Sample Name | Hydration Conditions | | | Carbonation Time (weeks) |
|---|---|---|---|---|
| | Salt(s) in Reaction | Time (min) | Final pH (average)* | |
| MgO_AA_Control | None | 30 | 10.685 ± 0.015 | 2 |
| 04182024_MgO_0p001MCaCl2 | 0.001 M $CaCl_2$ | 30 | 10.590 ± 0.031 | 2 |
| 04182024_MgO_0p01MCaCl2 | 0.010 M $CaCl_2$ | 30 | 10.806 ± 0.282 | 2 |
| 04182024_MgO_0p1MCaCl2 | 0.100 M $CaCl_2$ | 30 | 10.649 ± 0.0206 | 2 |
| 05302024_MgO_0p001MCaCl2 | 0.001M $CaCl_2$ | 30 | 10.769 ± 0.0571 | ND |
| 05302024_MgO_0p01MCaCl2 | 0.010 M $CaCl_2$ | 30 | 10.674 ± 0.0444 | ND |
| 03202024_MgO_0p03MCaCl2 | 0.030 M $CaCl_2$ | 30 | 10.312 ± 0.0584 | ND |
| 03202024_MgO_0p06MCaCl2 | 0.060 M $CaCl_2$ | 30 | 10.444 ± 0.0378 | ND |
| 05302024_MgO_0p1MCaCl2 | 0.100 M $CaCl_2$ | 30 | 10.741 ± 0.0667 | ND |
| 05072024_MgO_0p001MKCl | 0.001 M KCl | 30 | 10.648 ± 0.0489 | 2 |
| 05072024_MgO_0p01MKCl | 0.010 M KCl | 30 | 10.451 ± 0.144 | 2 |
| 05072024_MgO_0p1MKCl | 0.100 M KCl | 30 | 10.448 ± 0.0420 | 2 |
| 04302024_MgO_0p001MNaCl | 0.001 M NaCl | 30 | 10.873 ± 0.0473 | 2 |
| 04302024_MgO_0p01MNaCl | 0.010 M NaCl | 30 | 10.798 ± 0.132 | 2 |
| 04302024_MgO_0p1MNaCl | 0.100 M NaCl | 30 | 10.782 ± 0.0355 | 2 |
| 09232024_MgO_0p2MNaCl | 0.200 M NaCl | 30 | 11.113 ± 0.0277 | ND |
| 09232024_MgO_0p3MNaCl | 0.300 M NaCl | 30 | 11.0436 ±0.0055 | ND |
| 09232024_MgO_0p409M_NaCl | 0.409 M NaCl | 30 | 11.127 ± 0.0078 | ND |
| 05062024_MgO_0p485M_NaCl | 0.485 M NaCl | 30 | 10.820 ± 0.0806 | 2 |
| 06242024_MgO_0p409M_NaCl | 0.409 M NaCl | 30 | 10.659 ± 0.0376 | 2 |
| 06242024_MgO_NaCl_Na2SO4 | 0.409 M NaCl<br>0.0282 M $Na_2SO_4$ | 30 | 10.808 ± 0.080 | 2 |
| 06242024_MgO_NaCl_CaCl2 | 0.409 M NaCl<br>0.0103 M $CaCl_2$ | 30 | 10.573 ±0.044 | 2 |
| 06202024_MgO_NaCl_MgCl2 | 0.409 M NaCl<br>0.0523 M $MgCl_2$ | 30 | 10.133 ±0.059 | 2 |

ND = not done

*Averaged pH for the last 20 min of the 30 min reaction of MgO with aqueous salt solution.

## 5. pH Monitoring Results

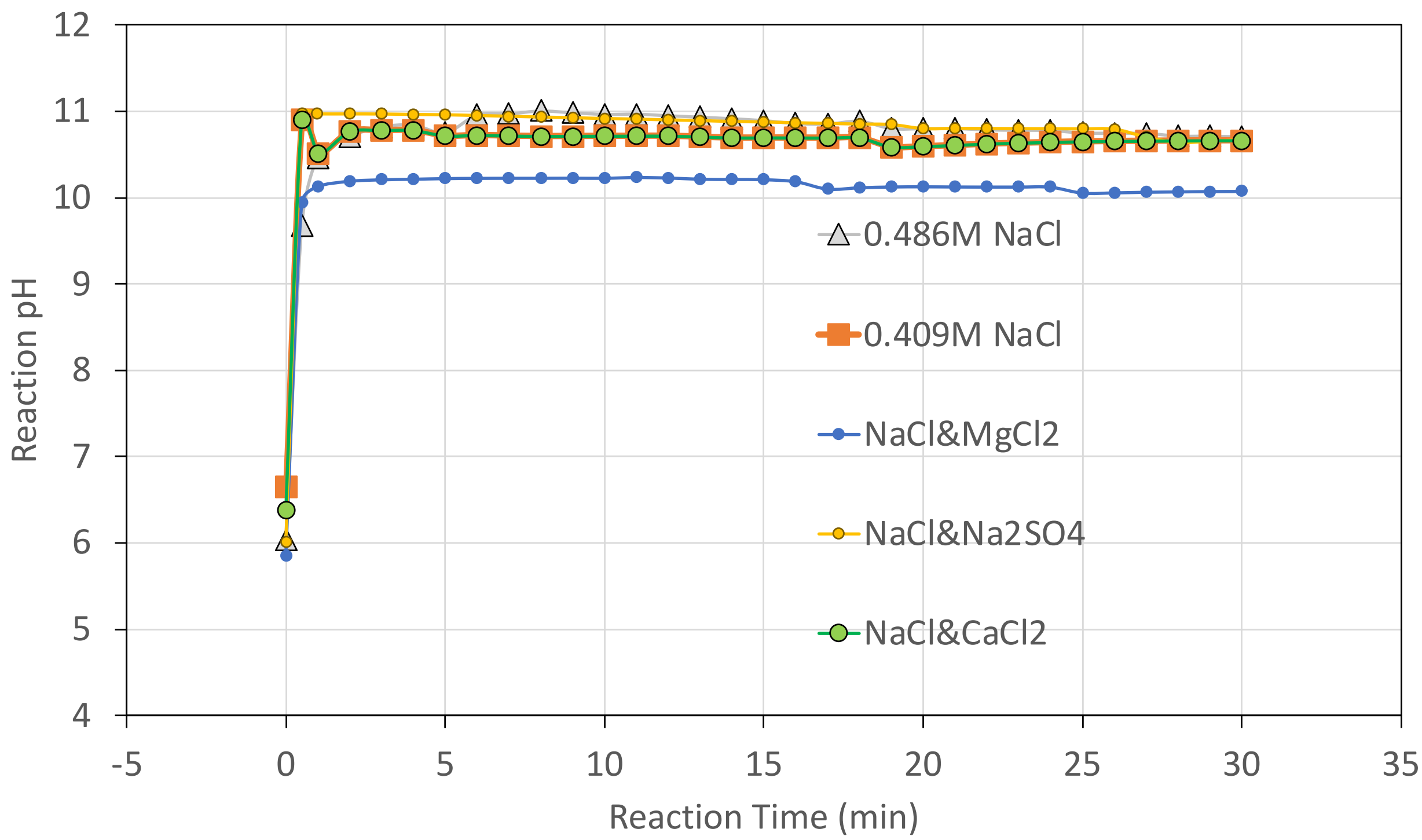


Figure S 1 Comparison of the pH changes during the 30 min time course of hydration of MgO in the presence of major component salts as binary mixtures with NaCl at their concentrations in seawater.

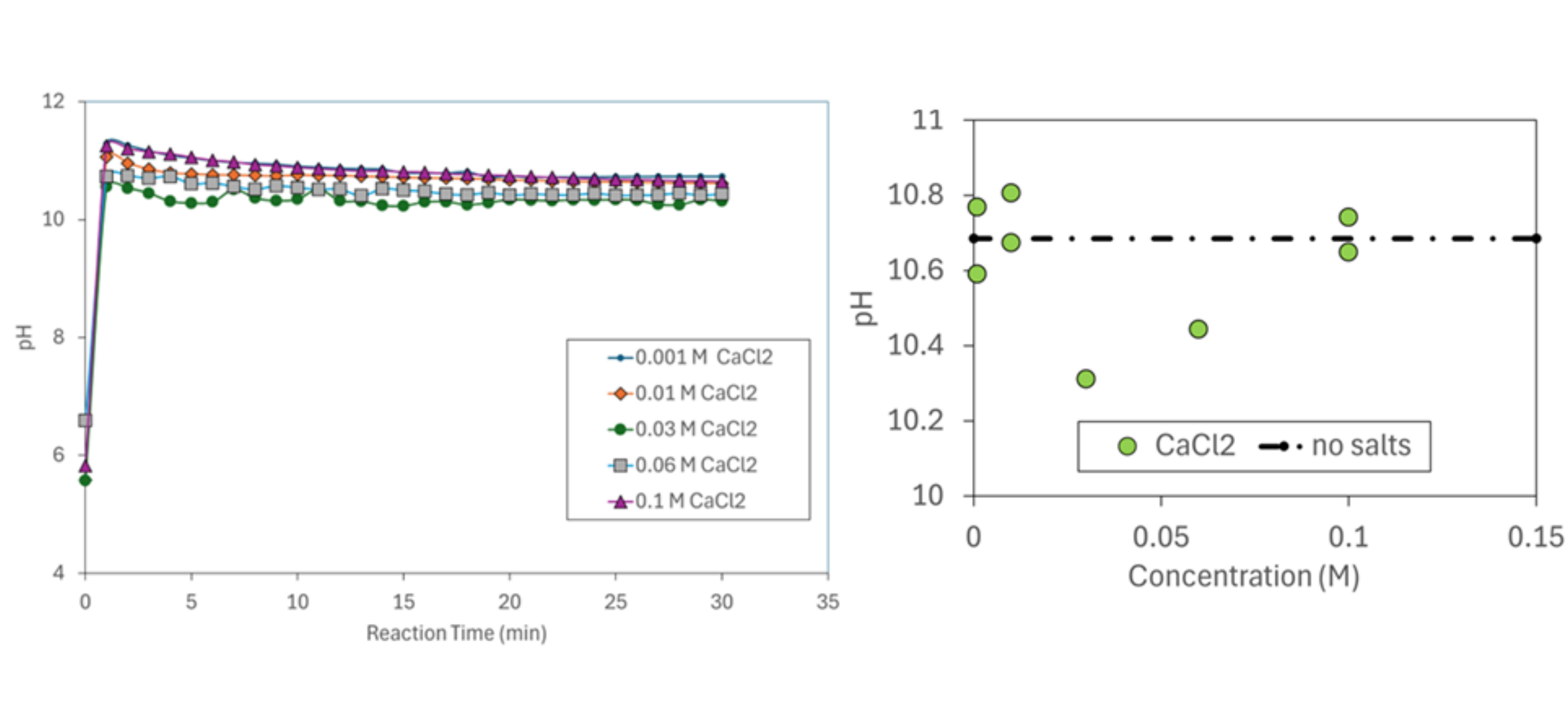


Figure S 2 The pH during the first 5 minutes of contact of MgO powder with $CaCl_2$ solution increases, followed by gradual decrease to a steady state during the final 20 minutes of the 30-

min reaction (left). Averages of the pH values for the last 20 minutes of reaction of MgO powder with 0.001 M and 0.100 M show a range of 10.3 – 10.8 (right). Dotted line marks the averaged pH values over the last 20 min of a 30-min control reaction of MgO powder with deionized water.

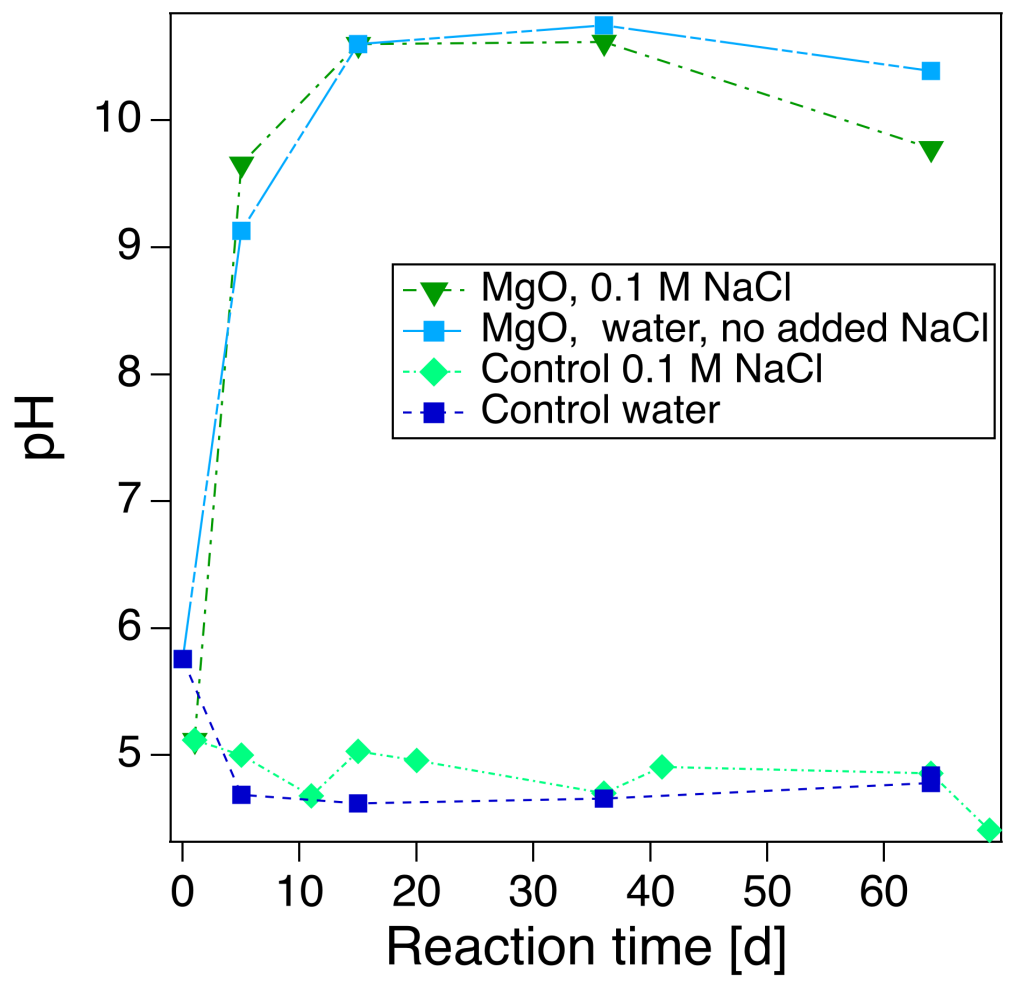


Figure S 3 The pH of 0.1 M NaCl in contact with MgO crystals increased rapidly and then stabilized. Final pH of 9.78 is slightly lower than for MgO in contact with water.

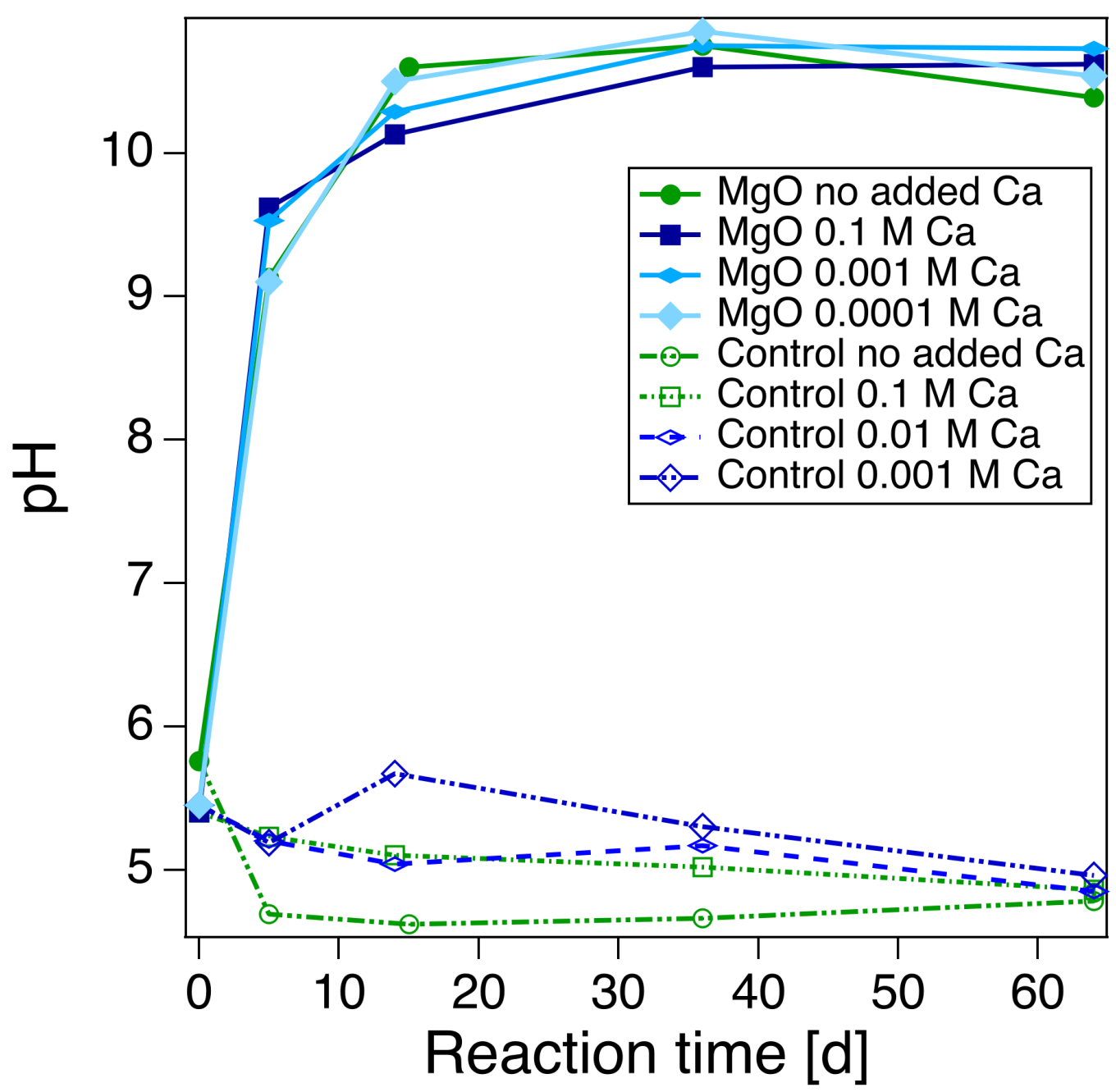


Figure S 4 The pH of $CaCl_2$ solutions in contact with MgO crystals increased during the first 35 days, then stabilized to 10.3-10.6 (a). The final pH values of MgO crystals reacted with $CaCl_2$ solutions were higher than that of MgO crystals incubated with deionized water (dotted line; b).

## 6. Additional ICP-OES Results

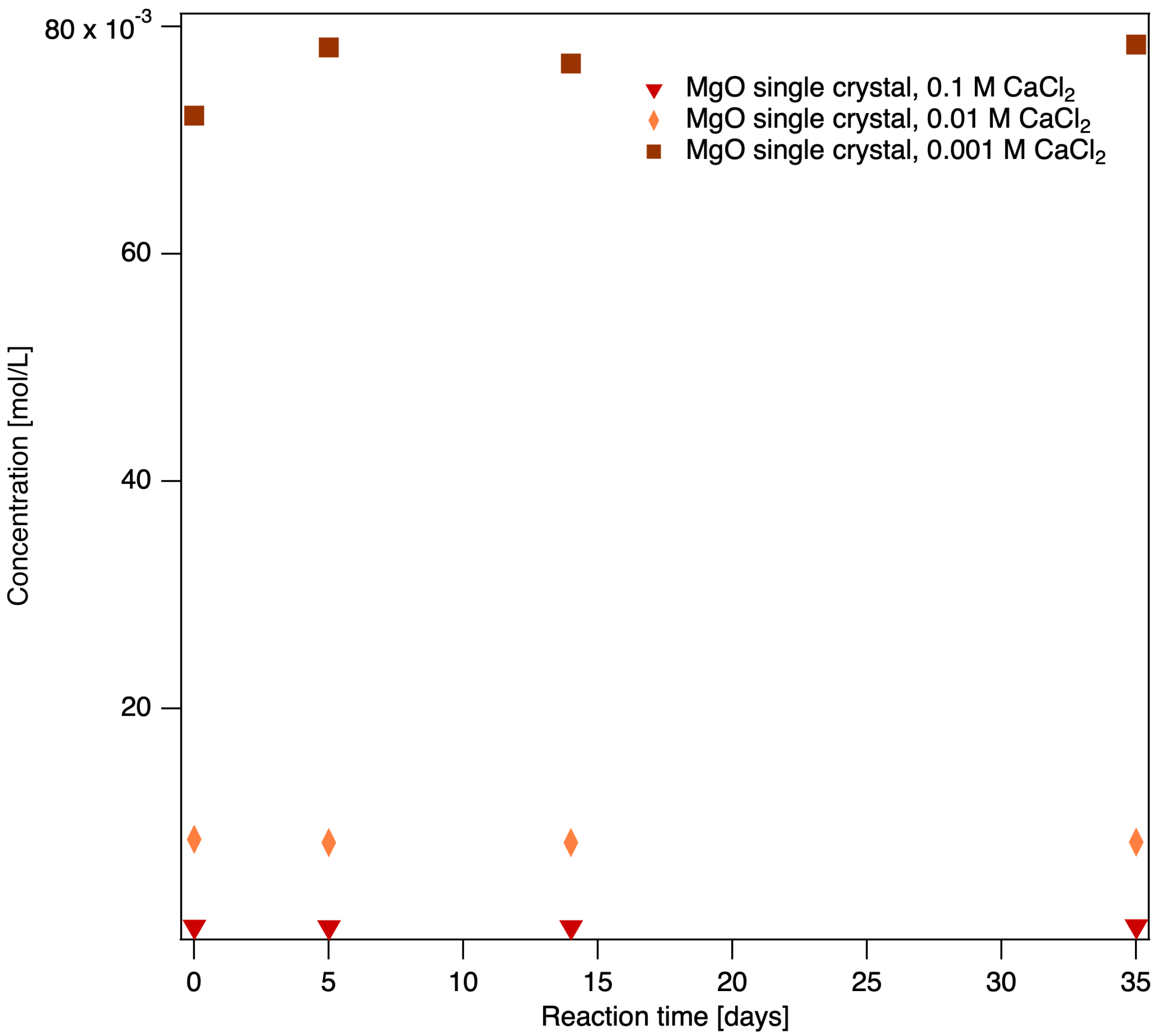


Figure S 5 Ca-concentration as a function of reaction time for MgO single crystals reacted in Ca-solutions. Ca-concentration remains stable.

## 7. Calculation of Dissolution Rate

To calculate the dissolution rate, the size of the MgO single crystals reacted with calcium chloride solutions was determined using caliper and the surface area was calculated. Averages of the measured surface area (Table S 5) were taken. Subsequently, a linear fit with intercept constraint of 0 (because at the beginning of the experiment, there is no Mg in the system) was performed to calculate the dissolution rate in mol/s. By multiplying with surface area, dissolution rates in mol $m^{-2}$ $s^{-1}$ were calculated.

Table S 6 Size measurements of MgO single crystals reacted with calcium chloride solutions.

| Experiment name | Height (mm) | Width (mm) | Length (mm) | surface area ($mm^2$) | | |
|---|---|---|---|---|---|---|
| MgO_0p1MCa_1 | 4.61 | 4.88 | 1.8 | 79.2 | | |
| MgO_0p1MCa_2 | 4.88 | 4.63 | 1.45 | 72.8 | | |
| MgO_0p1MCa_3 | 4.91 | 4.77 | 1.12 | 68.5 | Average ($mm^2$) | Standard deviation ($mm^2$) |
| MgO_0p1MCa_4 | 4.82 | 4.92 | 1.68 | 80.2 | 75.2 | 5.5 |
| | | | | | | |
| MgO_0p01MCa_1 | 4.81 | 4.72 | 1.39 | 71.9 | | |
| MgO_0p01MCa_2 | 4.69 | 4.83 | 1.31 | 70.2 | | |
| MgO_0p01MCa_3 | 4.98 | 4.82 | 1.26 | 72.7 | Average ($mm^2$) | Standard deviation ($mm^2$) |
| MgO_0p01MCa_4 | 4.89 | 4.75 | 1.53 | 76.0 | 72.7 | 2.4 |
| | | | | | | |
| MgO_0p001MCa_1 | 6.61 | 4.42 | 1.41 | 89.5 | | |
| MgO_0p001MCa_2 | 6.92 | 4.44 | 1.67 | 99.4 | | |
| MgO_0p001MCa_3 | 4.34 | 6.56 | 1.27 | 84.6 | Average ($mm^2$) | Standard deviation ($mm^2$) |
| MgO_0p001MCa_4 | 4.73 | 4.89 | 1.24 | 70.1 | 85.9 | 12.2 |

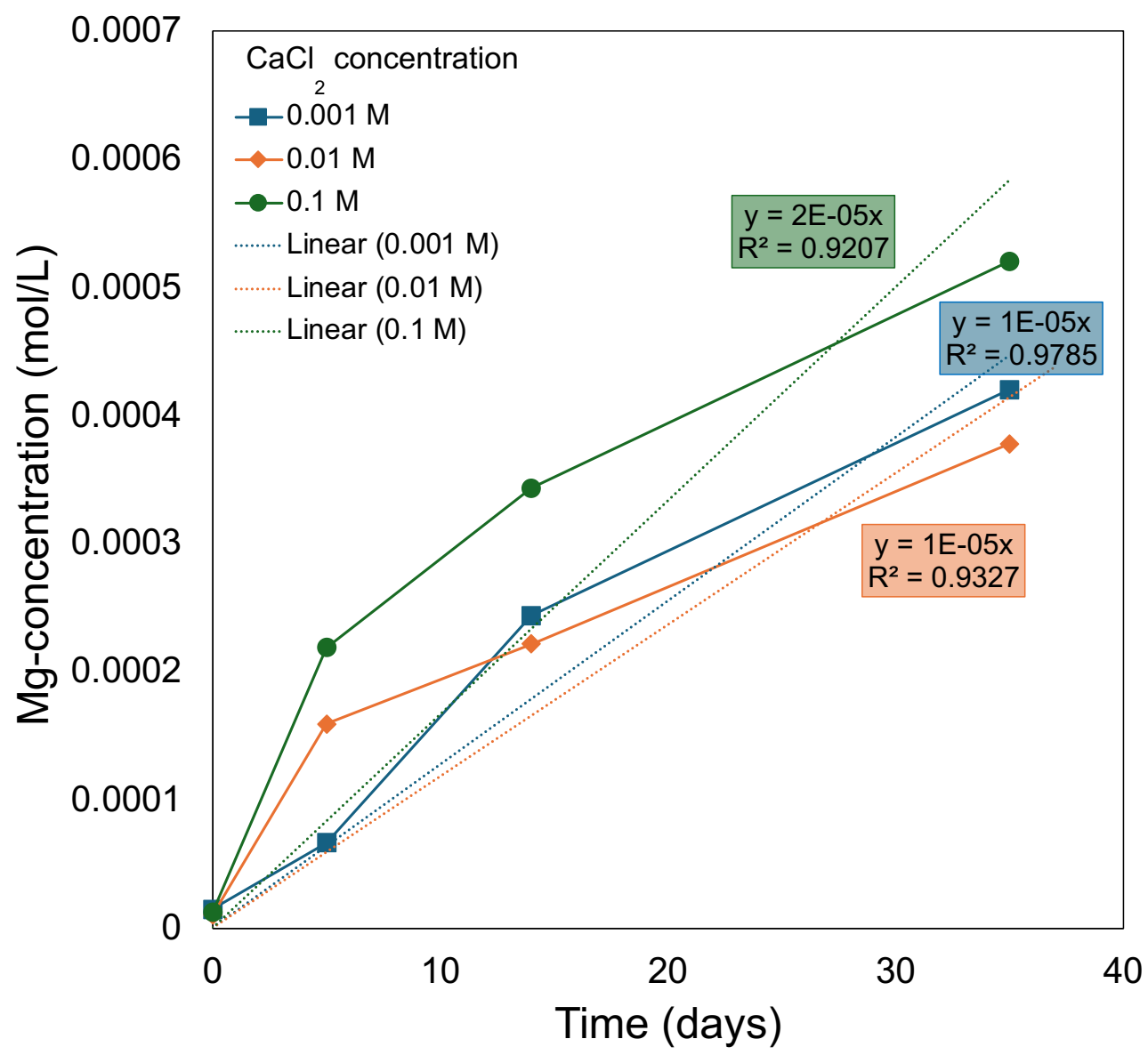


Figure S 6	Linear fit for Mg-concentrations measured by ICP-OES.

## 8. Additional Microscopy Results

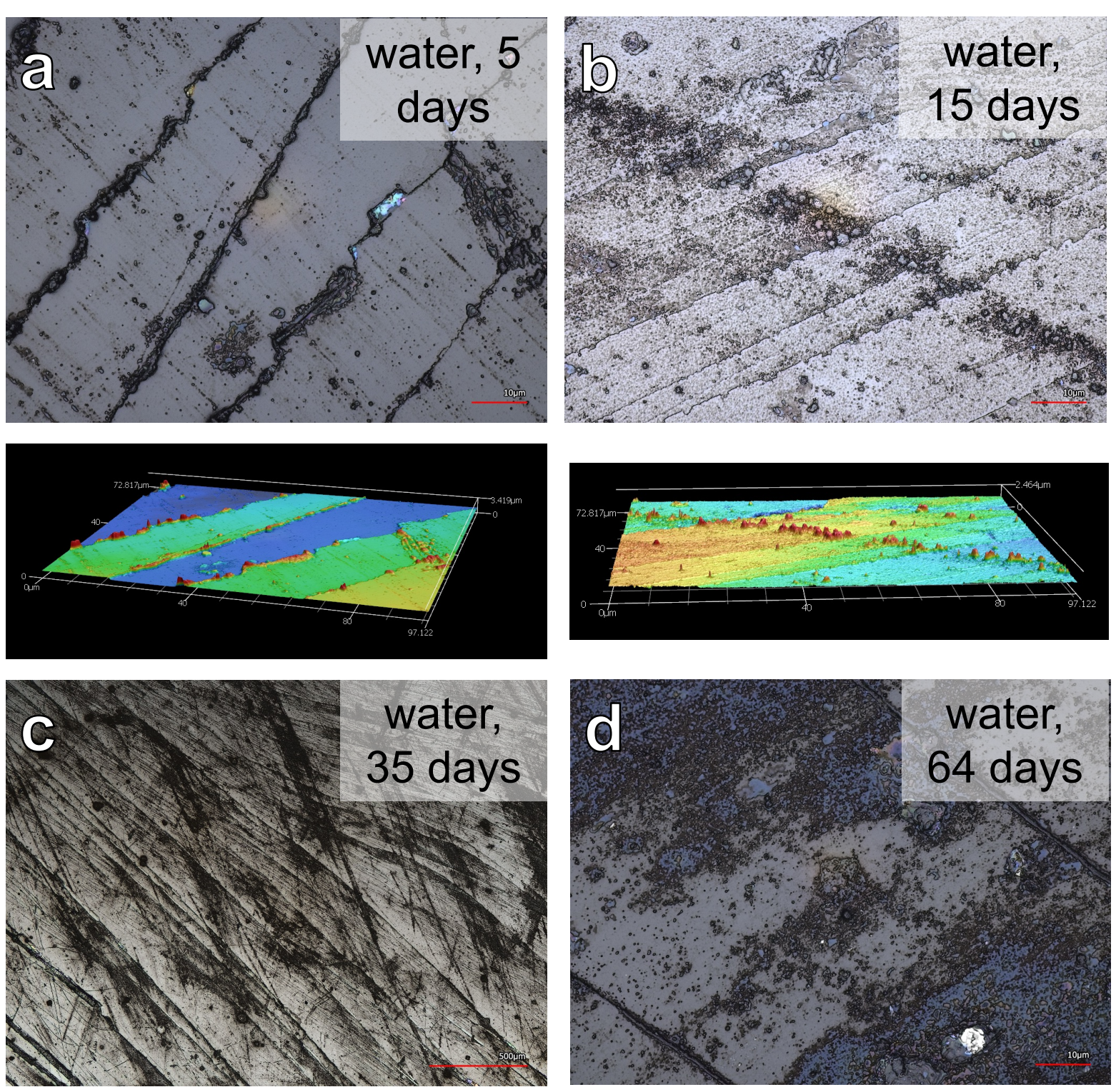


Figure S 7	Profilometer characterization of MgO crystals reacted with deionized water for 5-35 days. In comparison to the freshly cleaved MgO (Figure 1b, main manuscript), surfaces after 5 days (a), 15 days (b), 35 days (c) and 64 days (d) show secondary precipitation.

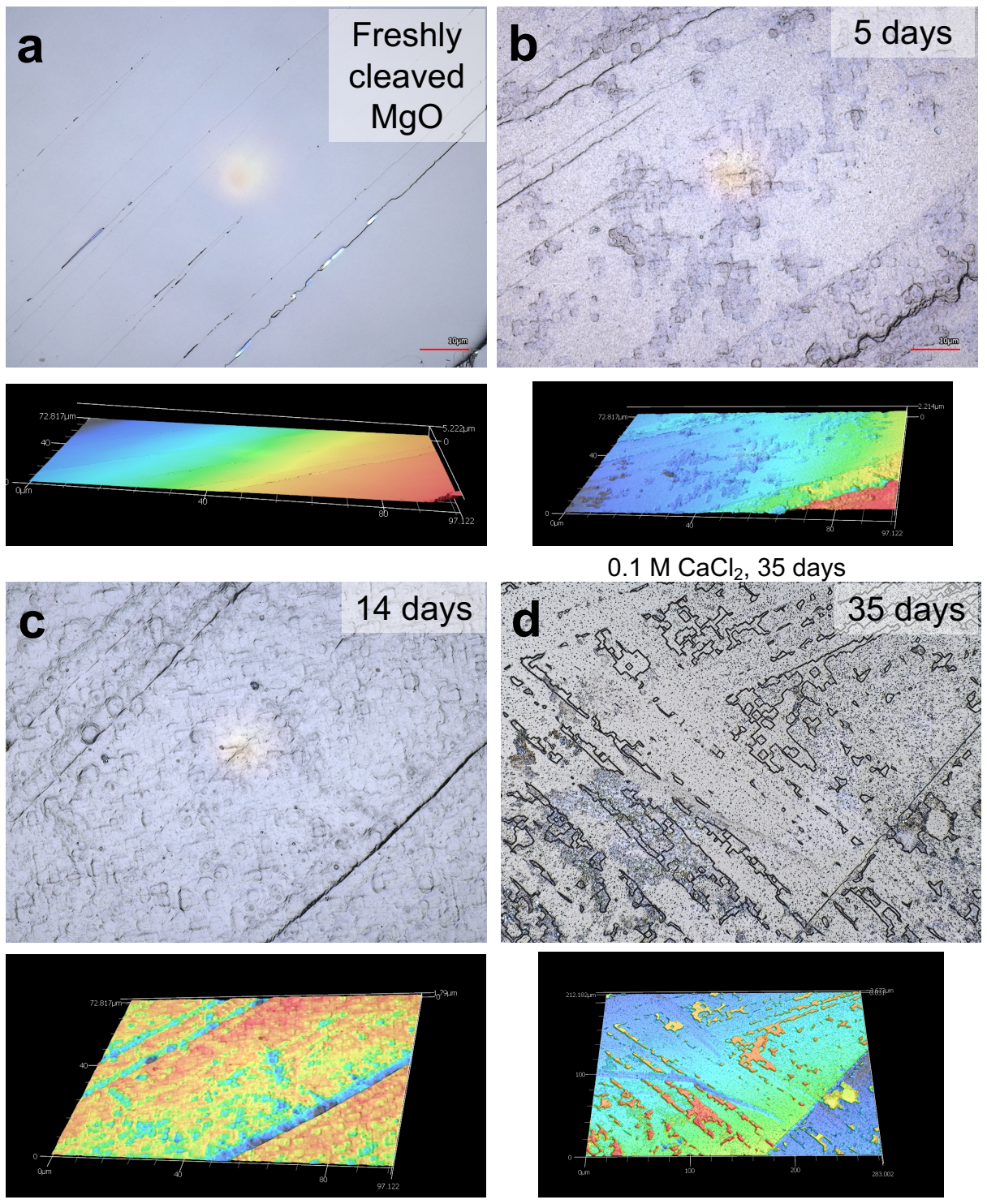


Figure S 8 Profilometer characterization of MgO crystals reacted with 0.1 M $CaCl_2$ for 5-35 days. In comparison to the freshly cleaved MgO (a), surfaces after 5 days (b), 14 days (c) and 35 days (d) show clear dissolution features.

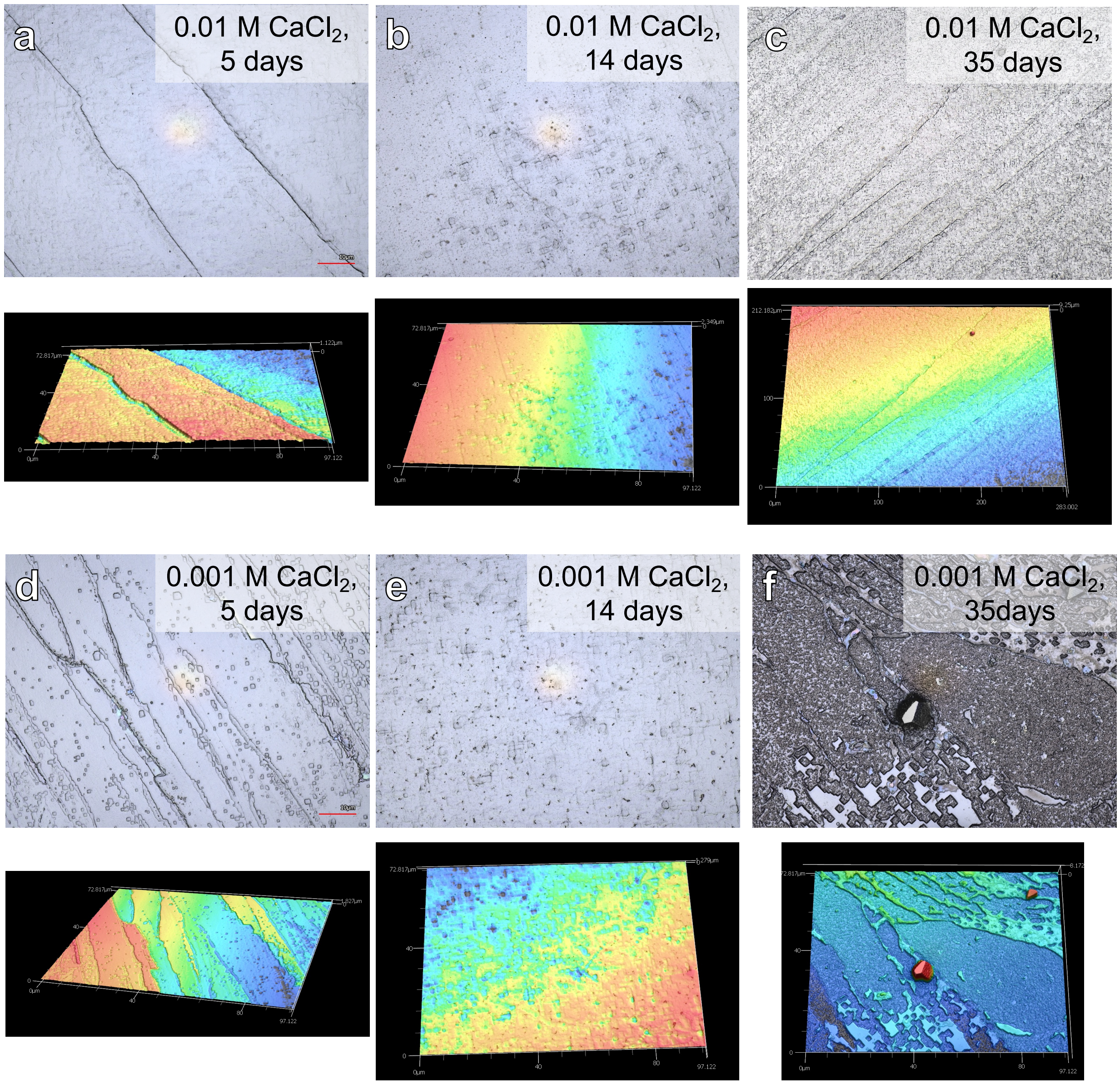


Figure S 9 Profilometer characterization of MgO crystals reacted with 0.01 M $CaCl_2$ (a-c) and 0.001 M $CaCl_2$ for 5-35 days (d-f). Dissolution features are visible after 5 days (a), 14 days (b) and 35 days (c) for 0.01 M $CaCl_2$. Newly formed precipitates are visible in (f).

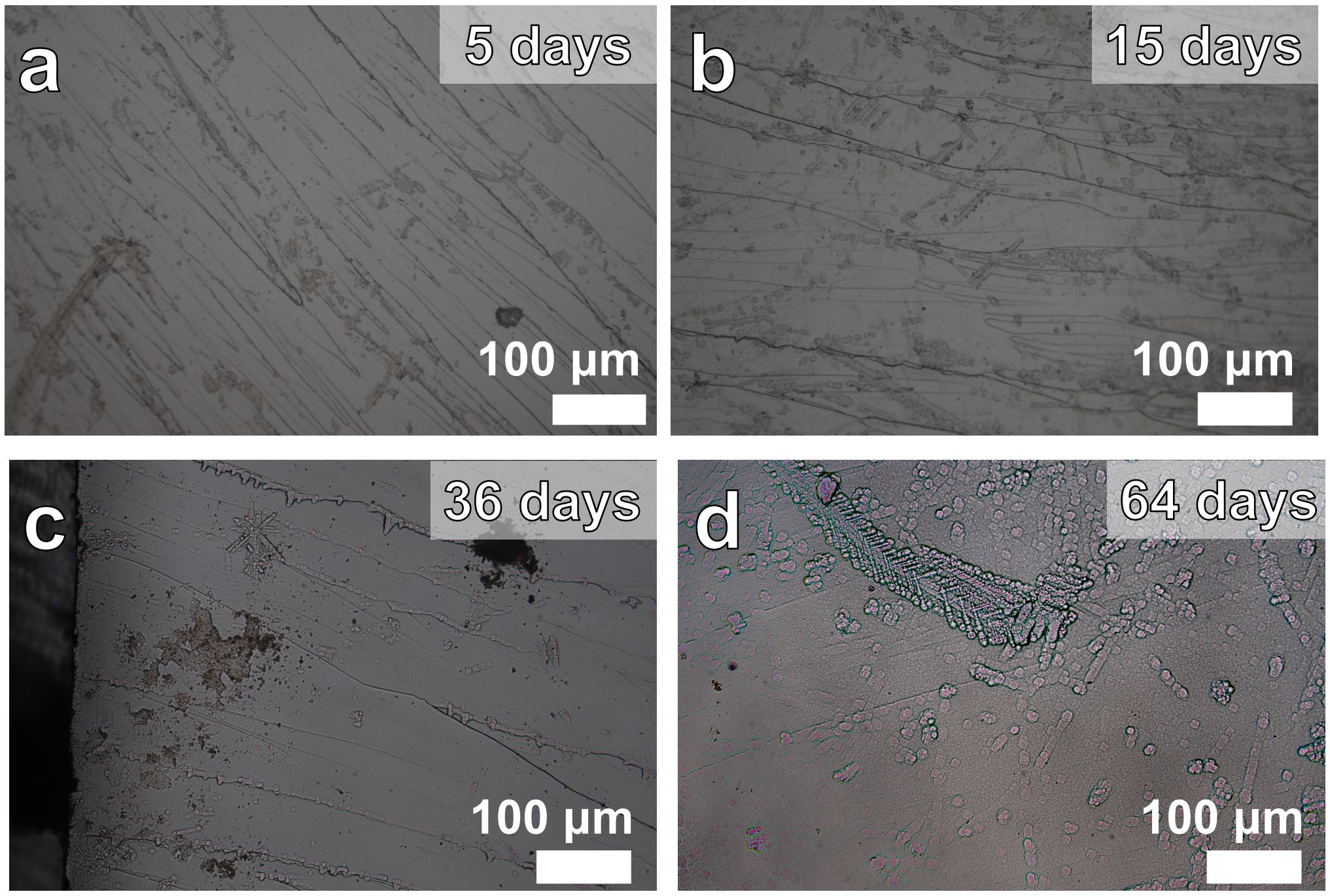


Figure S 10 Light microscopy imaging of MgO single crystal reaction with 0.1 M NaCl. a) 5 days of reaction, small precipitates are visible. b) 15 days of reaction, c) 36 days and d) 64 days with salt precipitates visible.

## 9. TGA-MS Results

Table S 7 TGA-MS results for $Mg(OH)_2$ samples following carbonation for 2 weeks. The $Mg(OH)_2$ samples were prepared by hydration of MgO powder for 30 min in salt solutions.

| Salt Solution used for Hydration | Mass reduction (%) | Mass loss (%) |
|---|---|---|
| 0.001 M $CaCl_2$ | 75.068 | 24.932 |
| 0.010 M $CaCl_2$ | 73.434 | 26.566 |
| 0.100 M $CaCl_2$ | 75.825 | 24.175 |
| 0.001 M KCl | 77.963 | 22.037 |
| 0.010 M KCl | 75.772 | 24.228 |
| 0.100 M KCl | 73.940 | 26.060 |
| 0.001 M NaCl | 76.112 | 23.888 |
| 0.010 M NaCl | 74.880 | 25.120 |
| 0.1 M NaCl | 68.870 | 31.130 |

| | | |
|---|---|---|
| 0.409 M NaCl | 57.813 | 42.187 |
| 0.409 M NaCl, 0.0103 M $CaCl_2$ | 72.718 | 27.282 |
| 0.409 M NaCl, 0.0532 M $MgCl_2$ | 63.060 | 36.940 |
| 0.409 M NaCl, 0.028 M $Na_2SO_4$ | 67.228 | 32.772 |

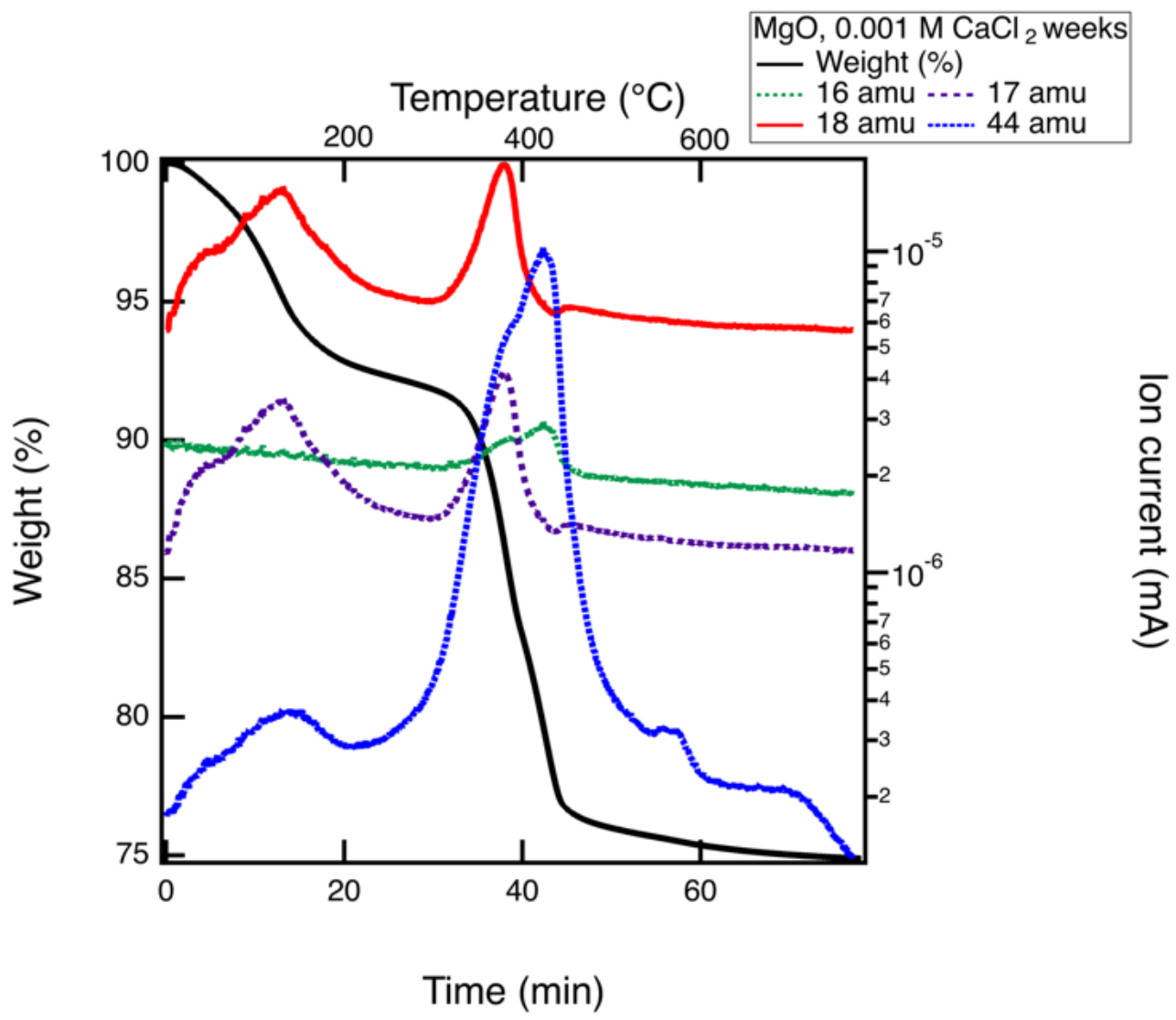


Figure S 11. TGA-MS of MgO powder hydrated with 1 mM $CaCl_2$ after carbonation for 2 weeks.

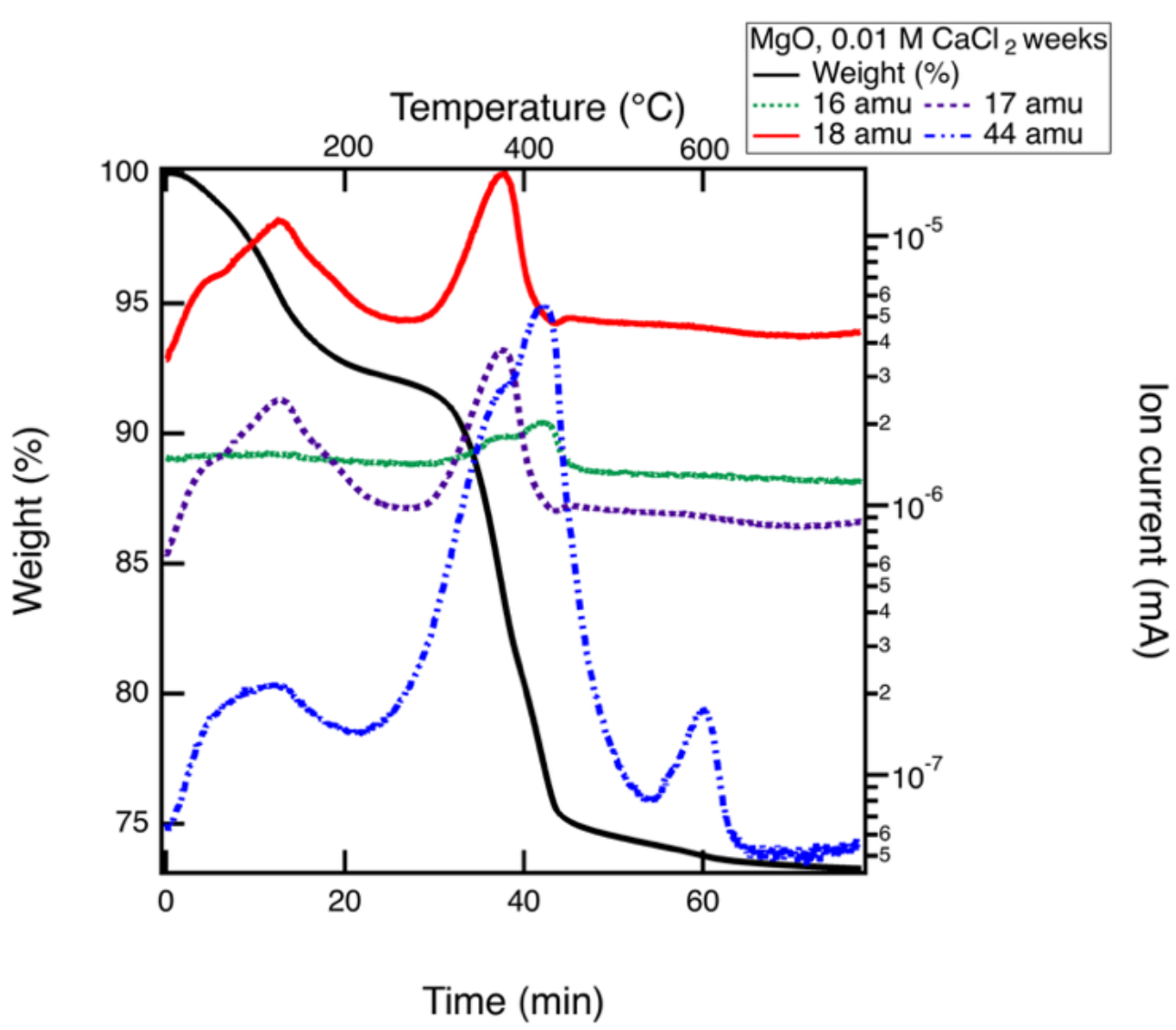


Figure S12. TGA-MS of MgO powder hydrated with 10 mM $CaCl_2$ after carbonation for 2 weeks.

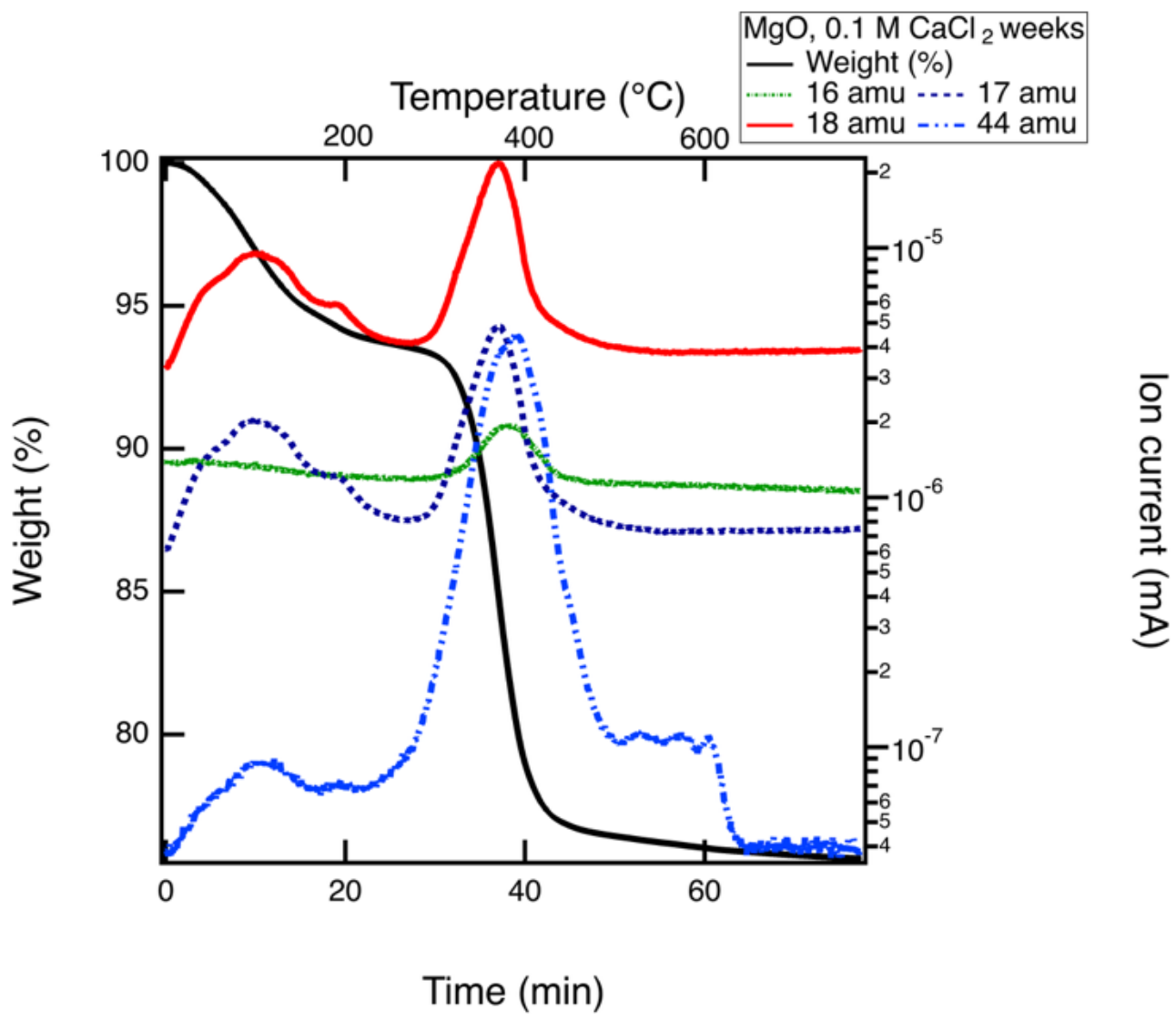


Figure S13. TGA-MS of MgO powder hydrated with 100 mM $CaCl_2$ after carbonation for 2 weeks.

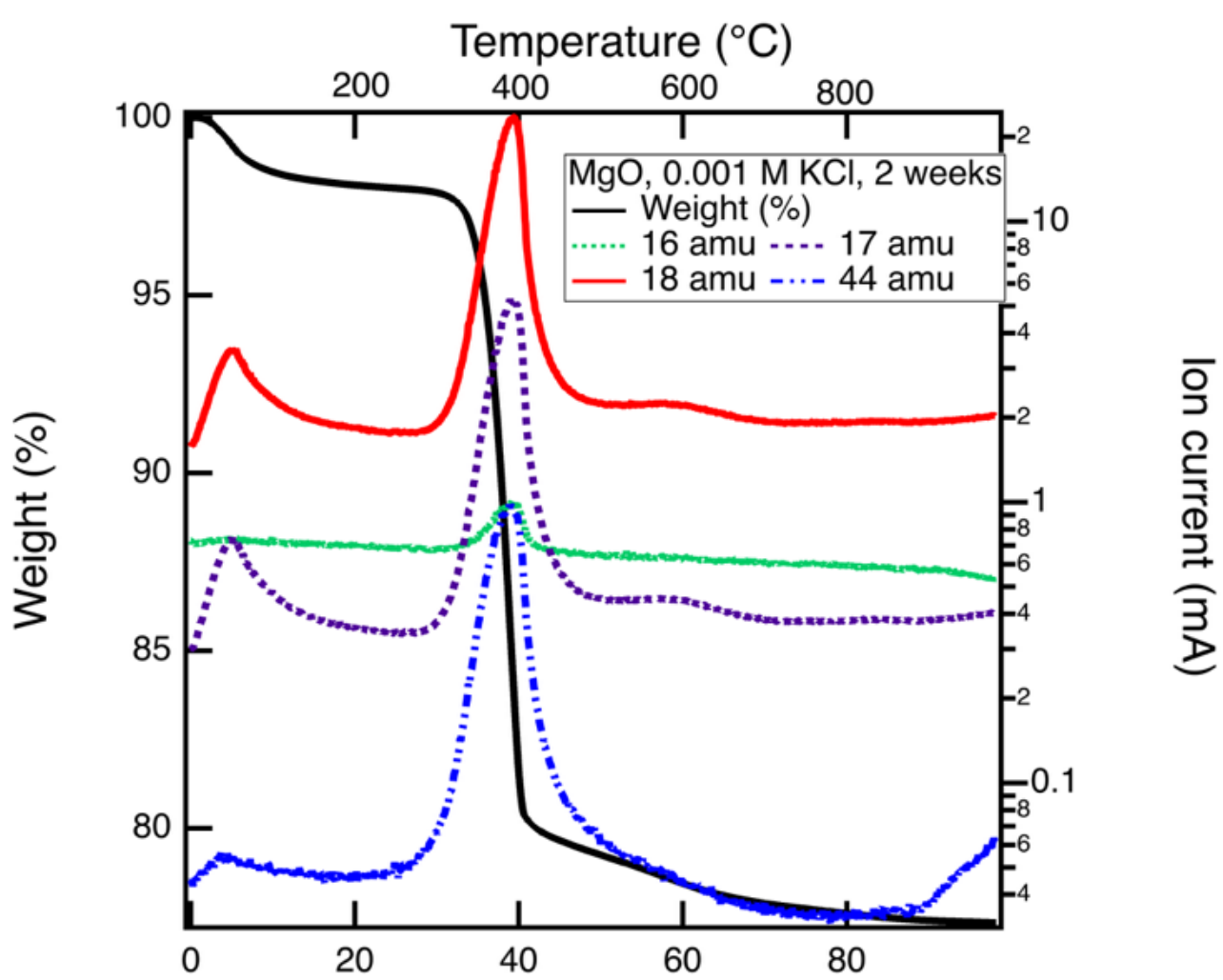


Figure S14. TGA-MS of MgO powder hydrated with 1 mM KCl after carbonation for 2 weeks.

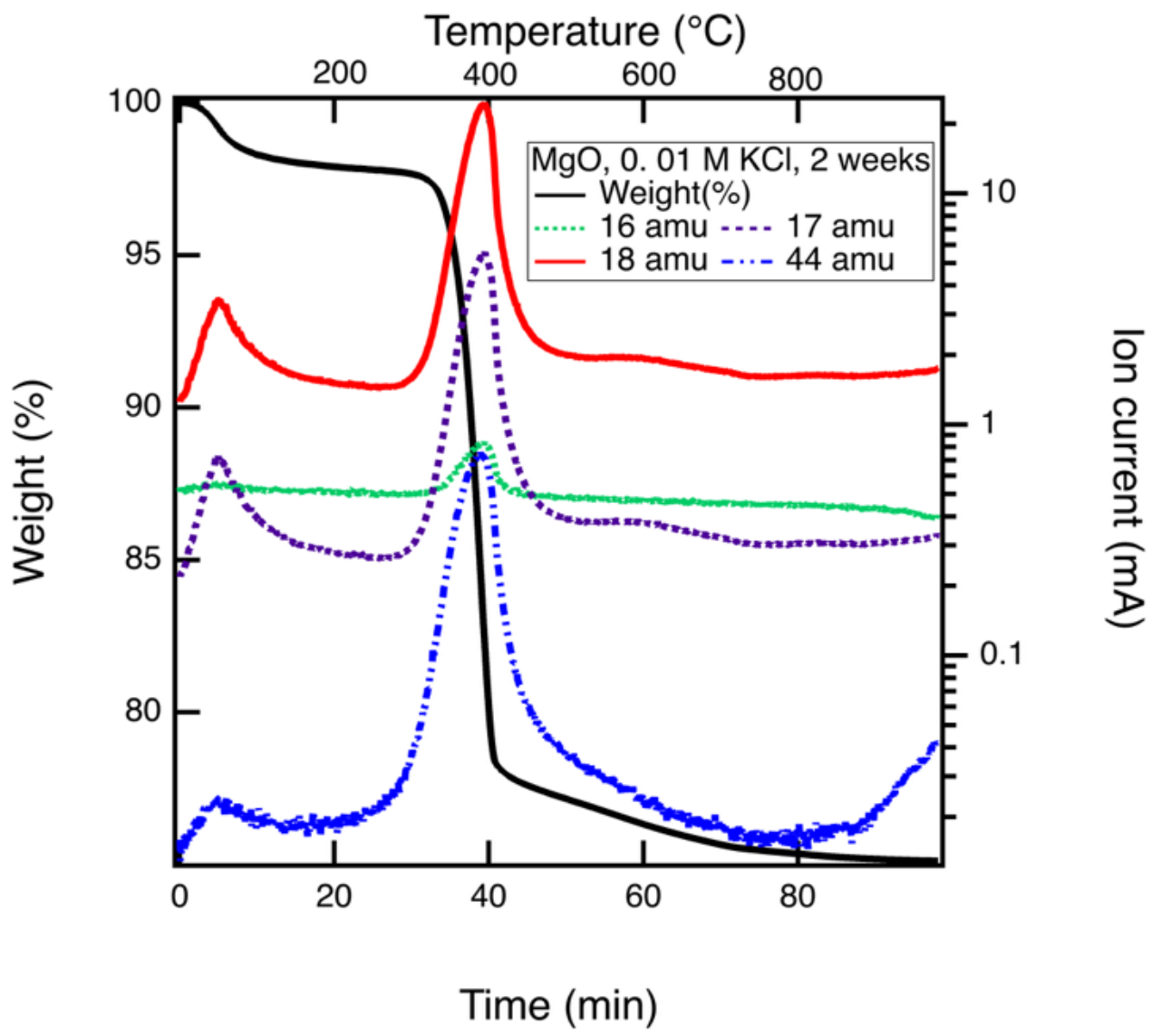


Figure S15. TGA-MS of MgO powder hydrated with 10 mM KCl after carbonation for 2 weeks.

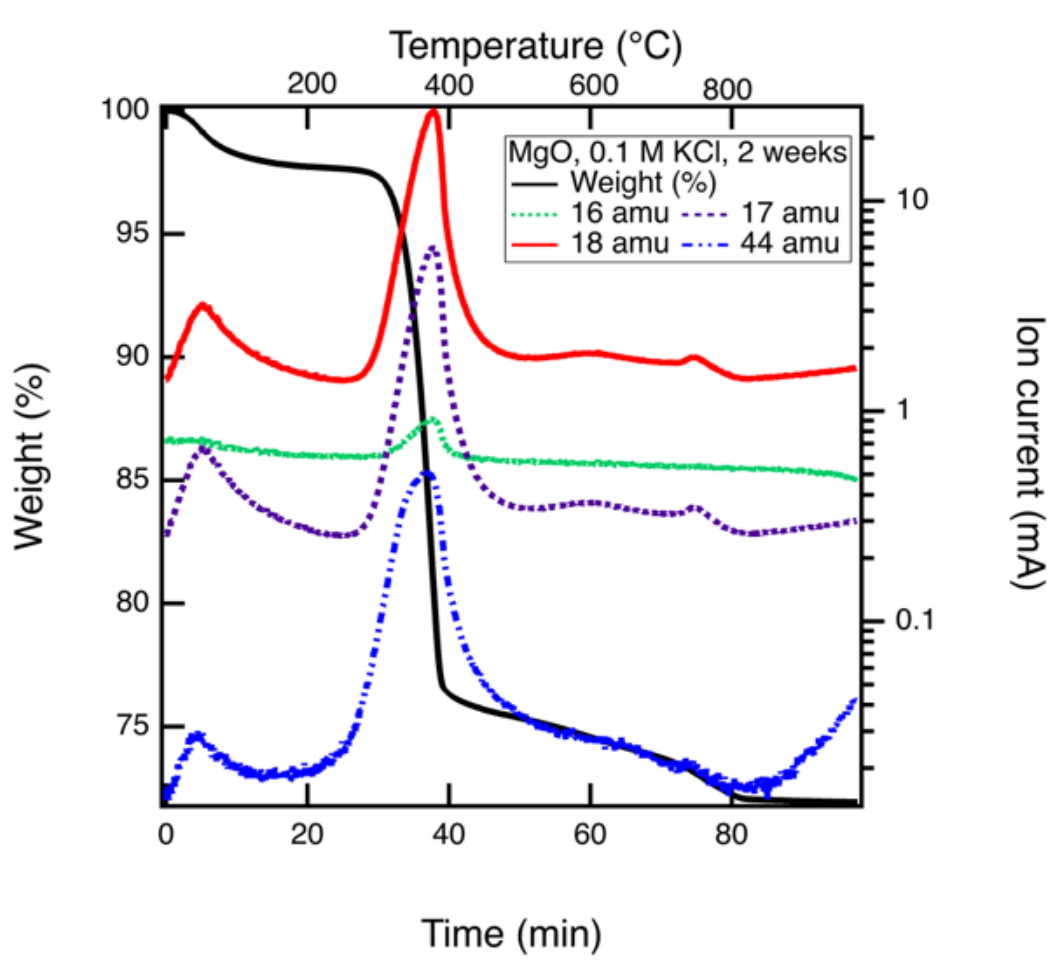


Figure S16. TGA-MS of MgO powder hydrated with 100 mM KCl after carbonation for 2 weeks.

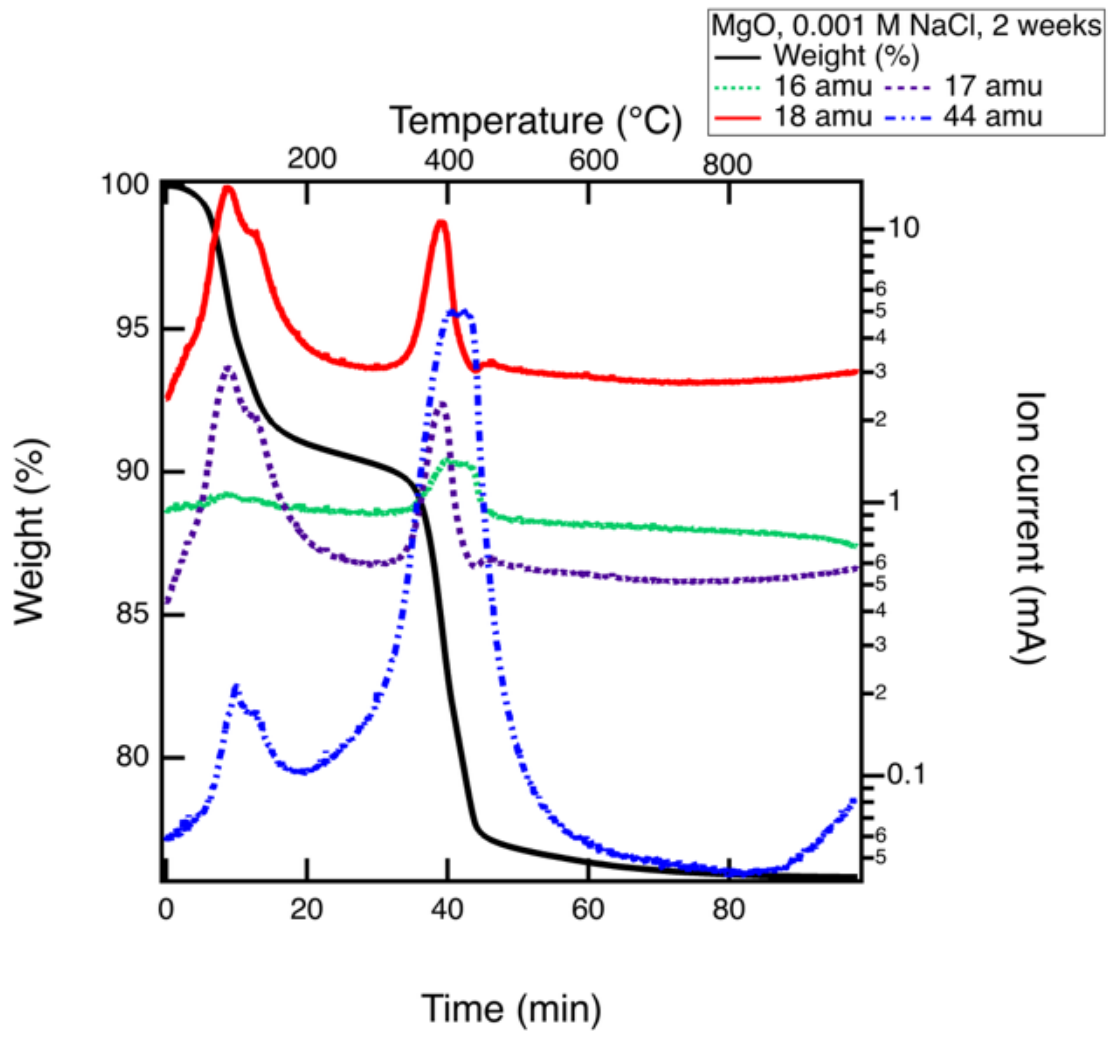


Figure S17. TGA-MS of MgO powder hydrated with 1 mM NaCl after carbonation for 2 weeks.

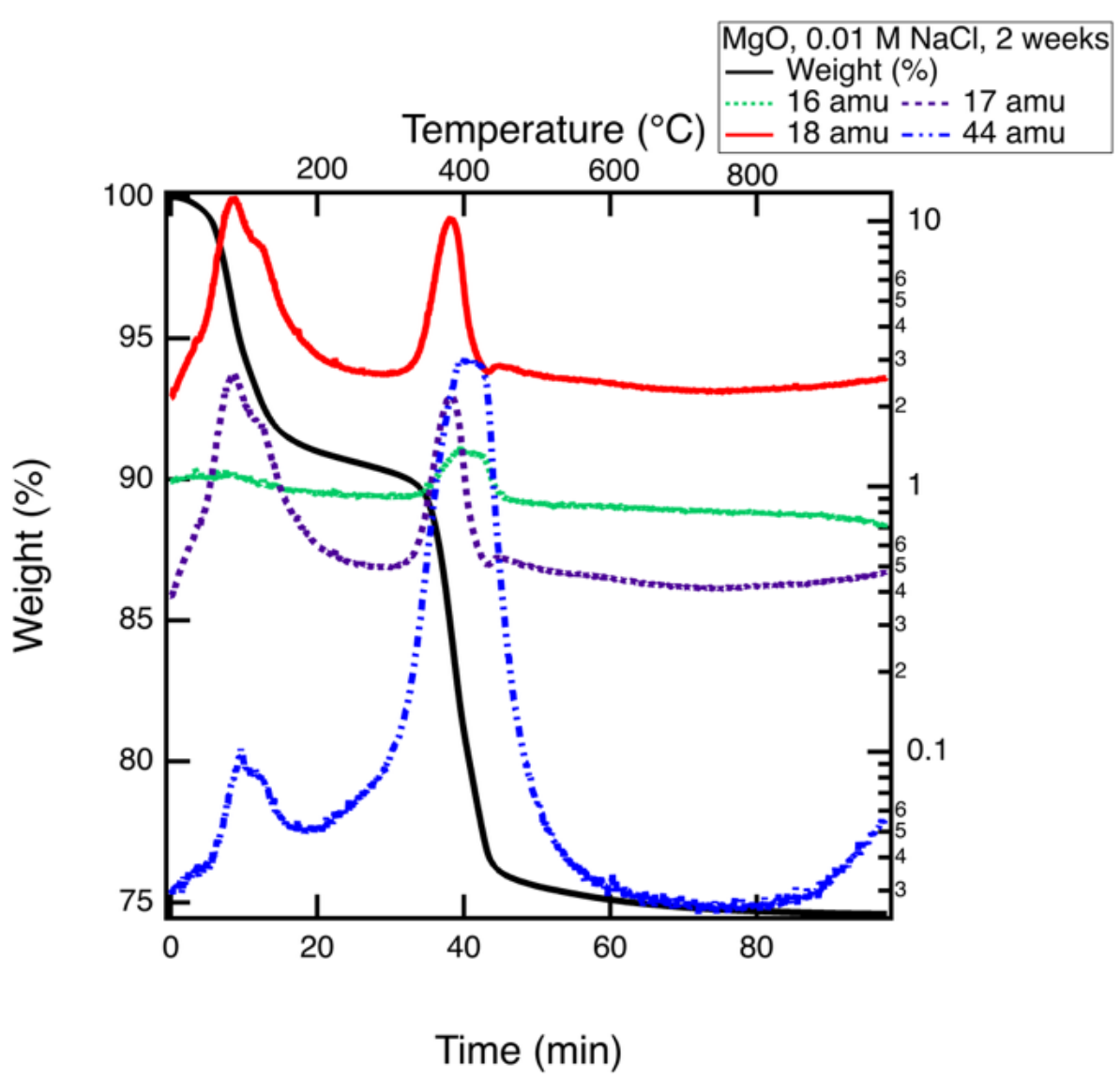


Figure S18. TGA-MS of MgO powder hydrated with 10 mM NaCl after carbonation for 2 weeks.

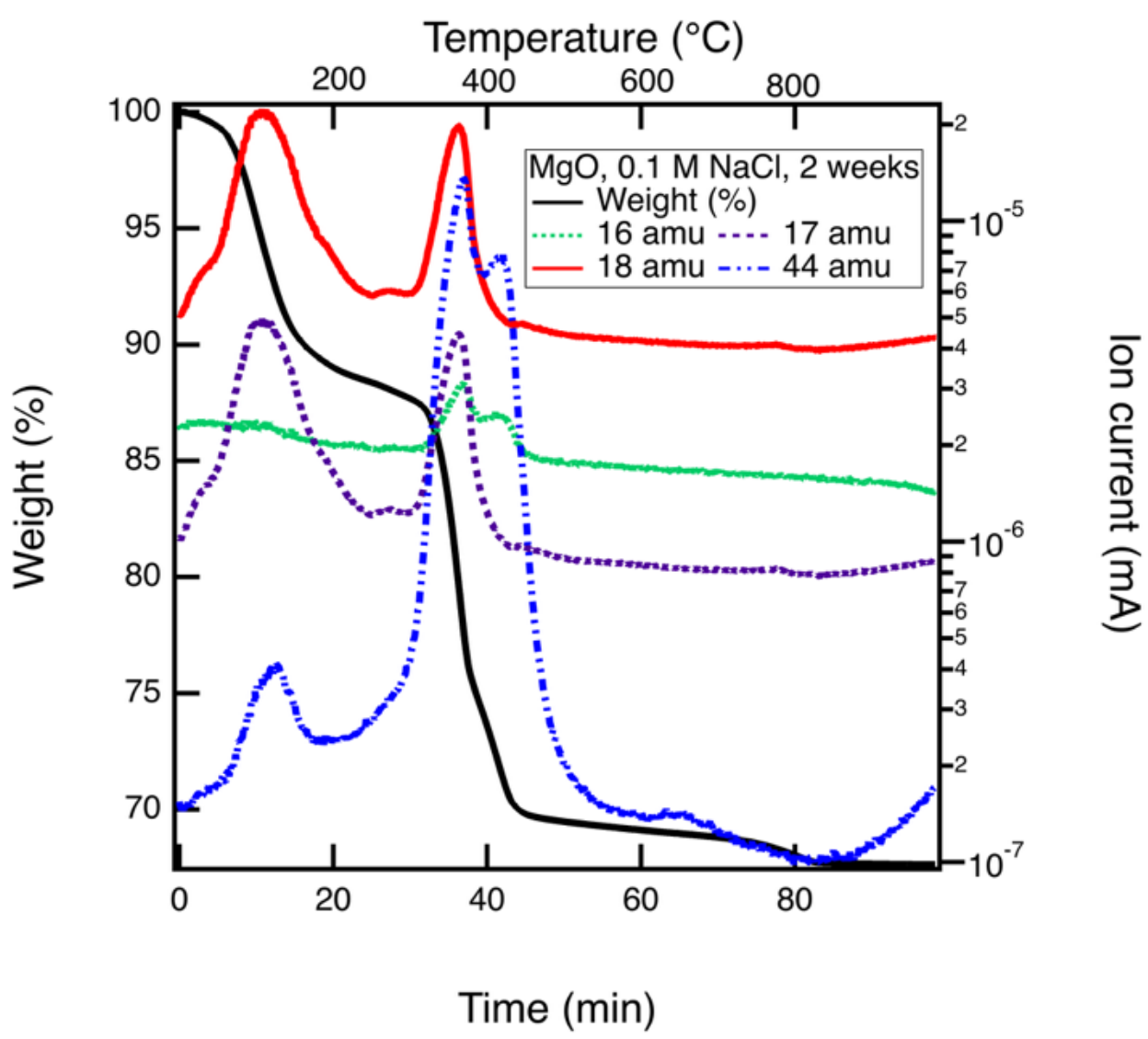


Figure S19. TGA-MS of MgO powder hydrated with 100 mM NaCl after carbonation for 2 weeks.

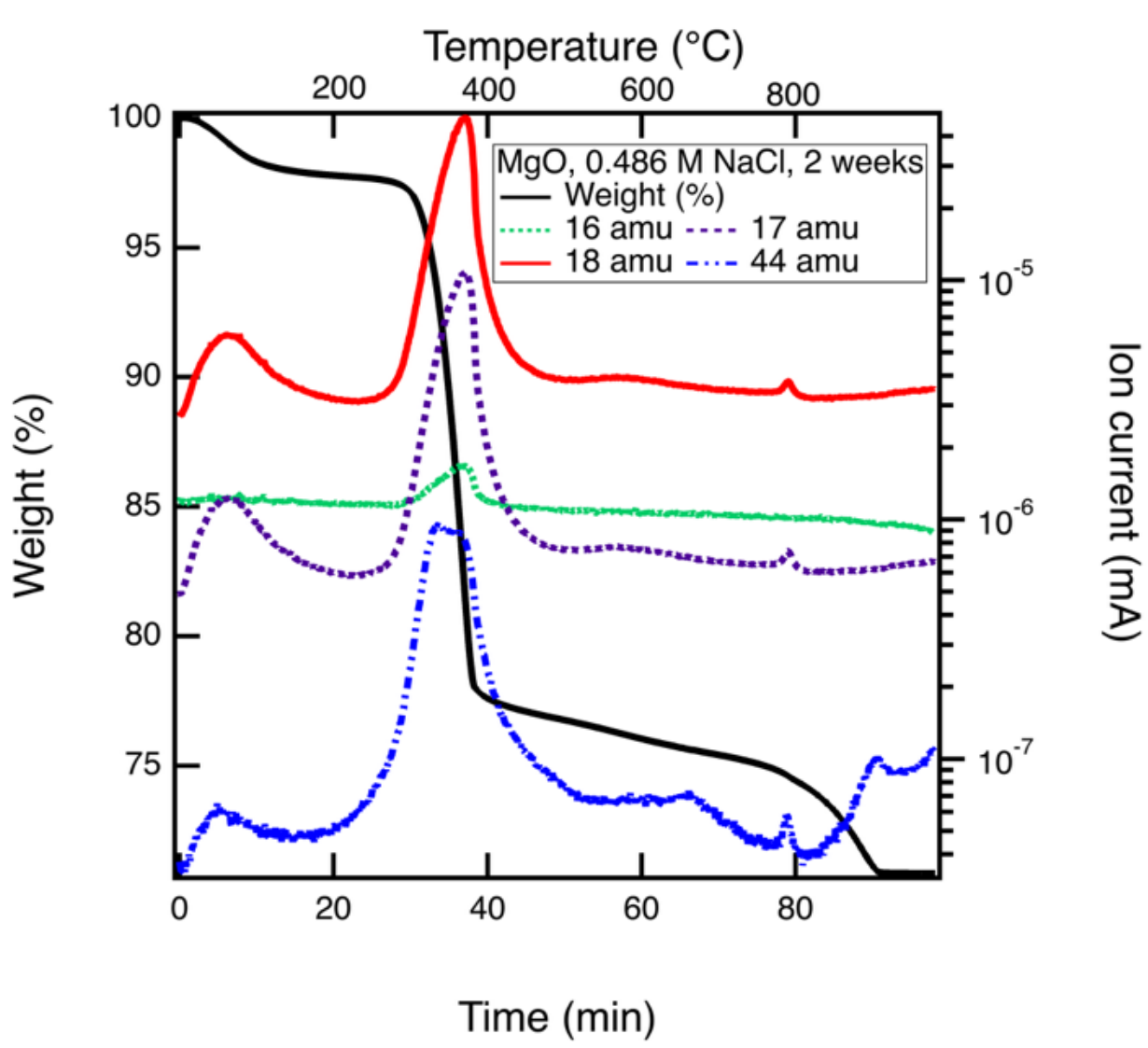


Figure S 20. TGA-MS of MgO powder hydrated with 486 mM NaCl after carbonation for 2 weeks.

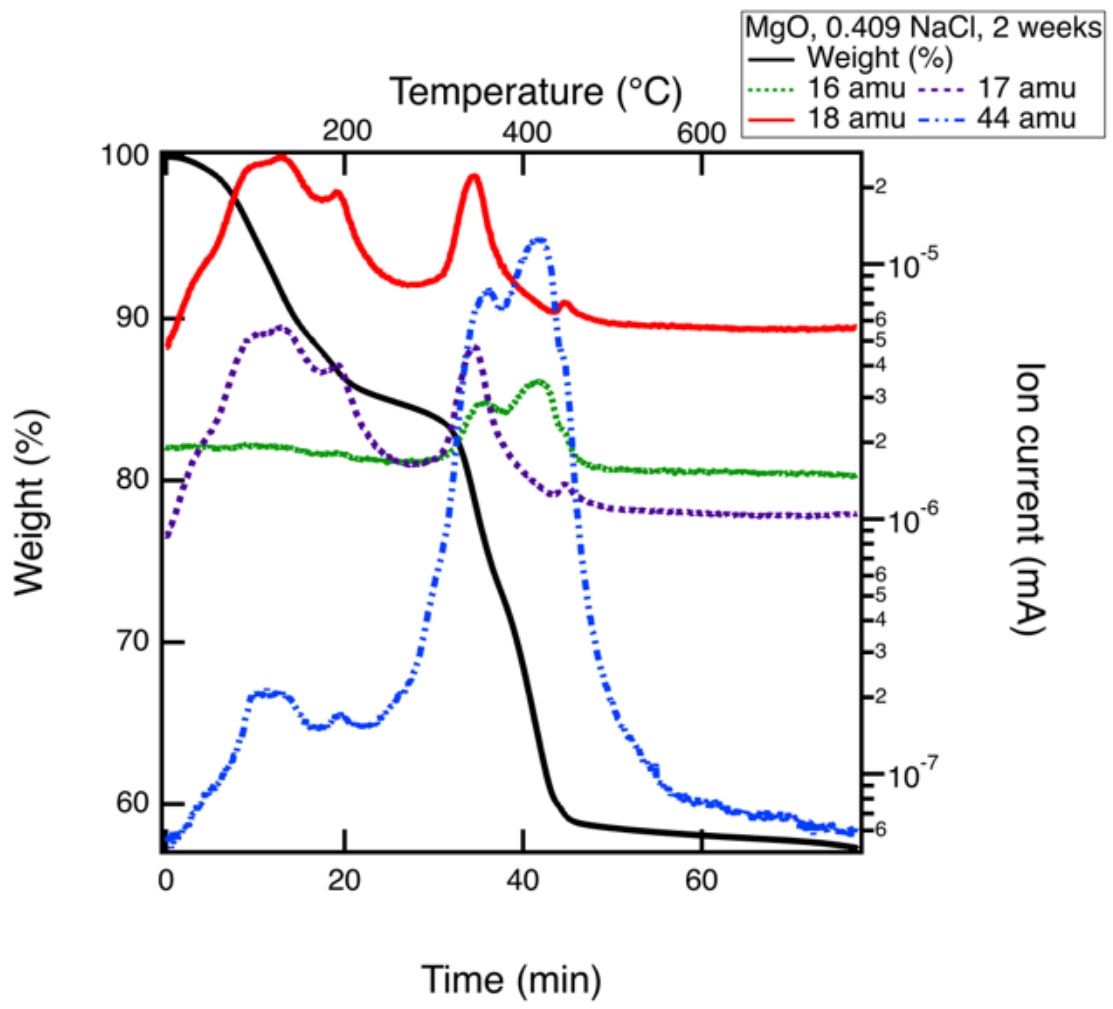


Figure S 21. TGA-MS of MgO powder hydrated with 409 mM NaCl after carbonation for 2 weeks.

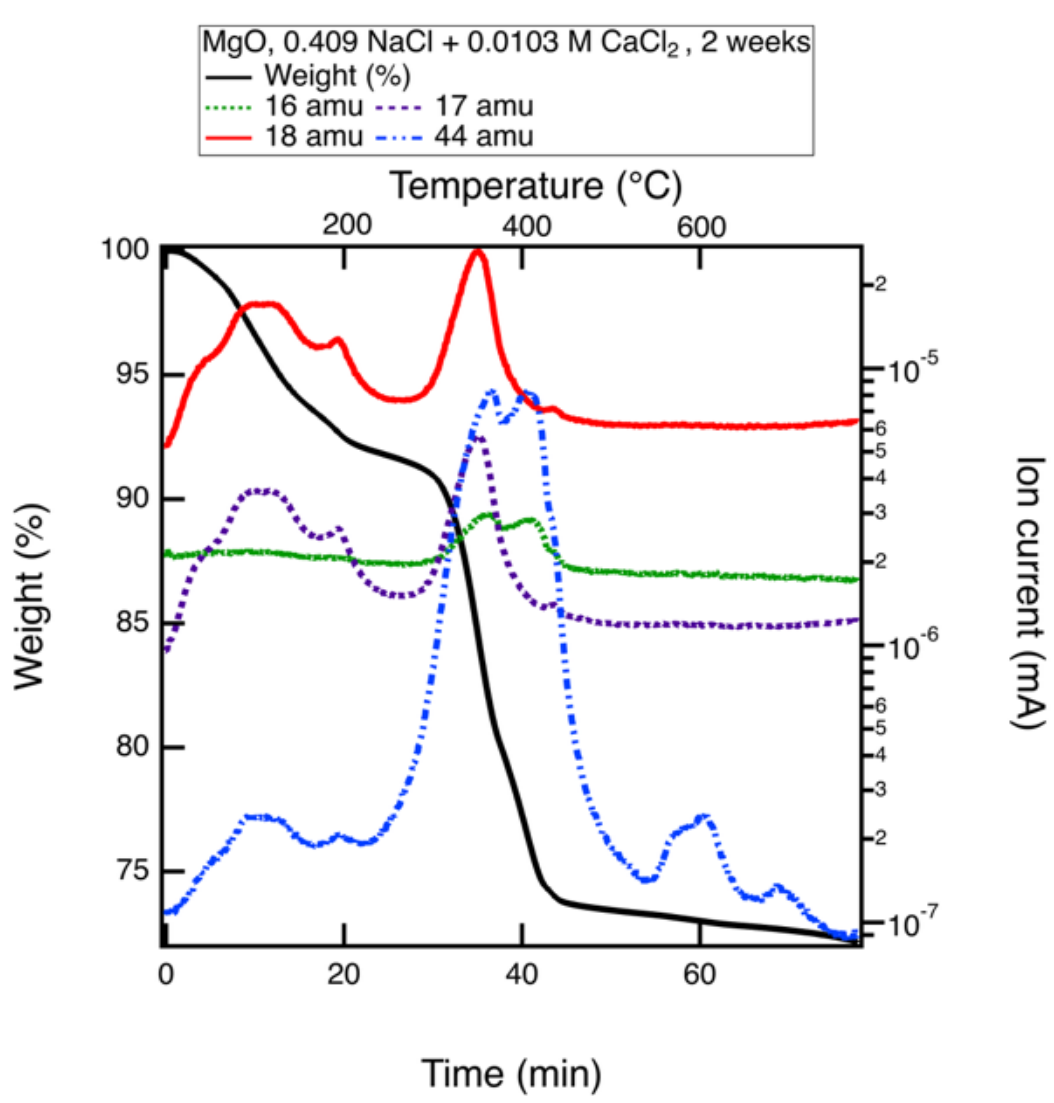


Figure S 22. TGA-MS of MgO powder hydrated with 409 mM NaCl and 10.3 mM $CaCl_2$ after carbonation for 2 weeks. Heating rate 10 °C $min^{-1}$.

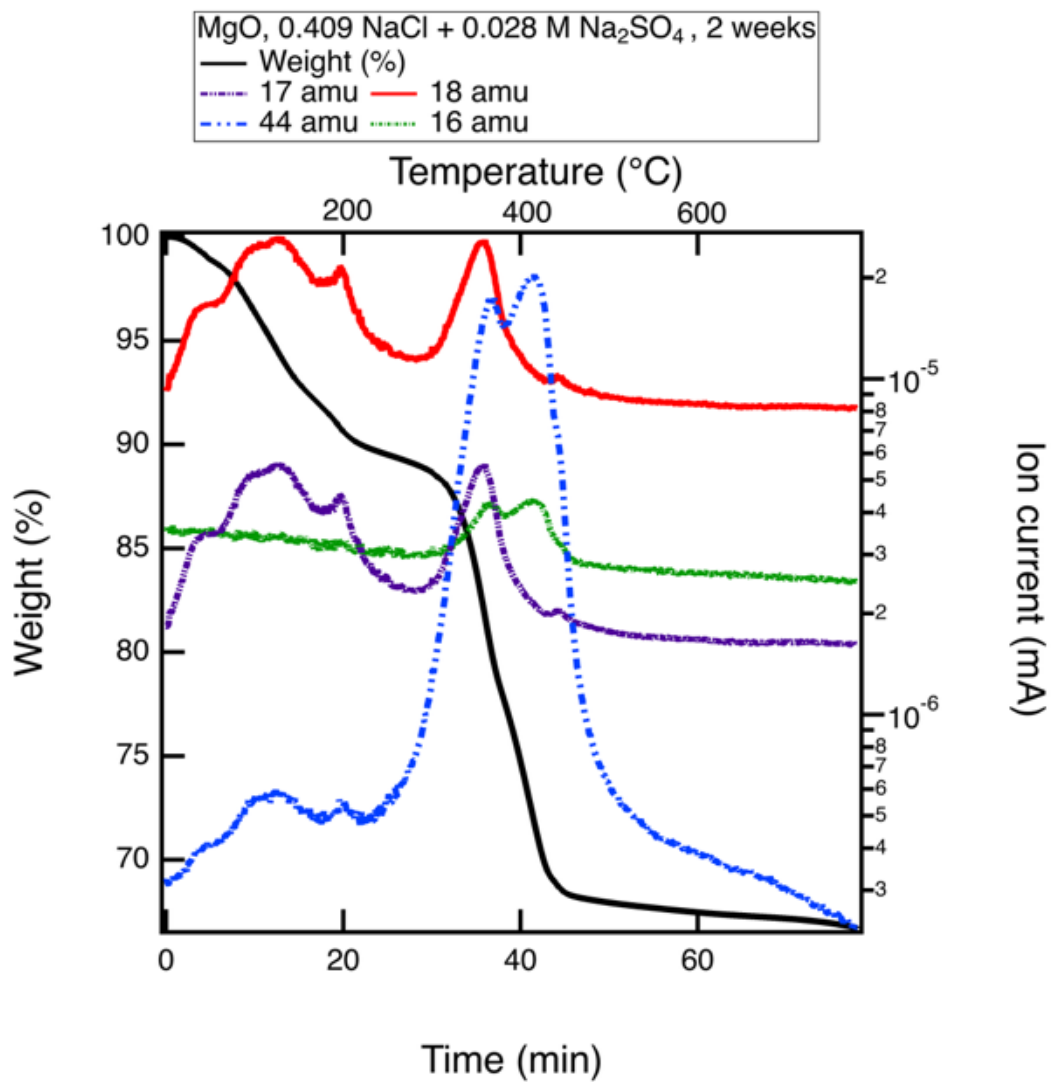


Figure S23. TGA-MS of MgO powder hydrated with 409 mM NaCl and 28.2 mM $Na_2SO_4$ after carbonation for 2 weeks. Heating rate 10 °C $min^{-1}$.

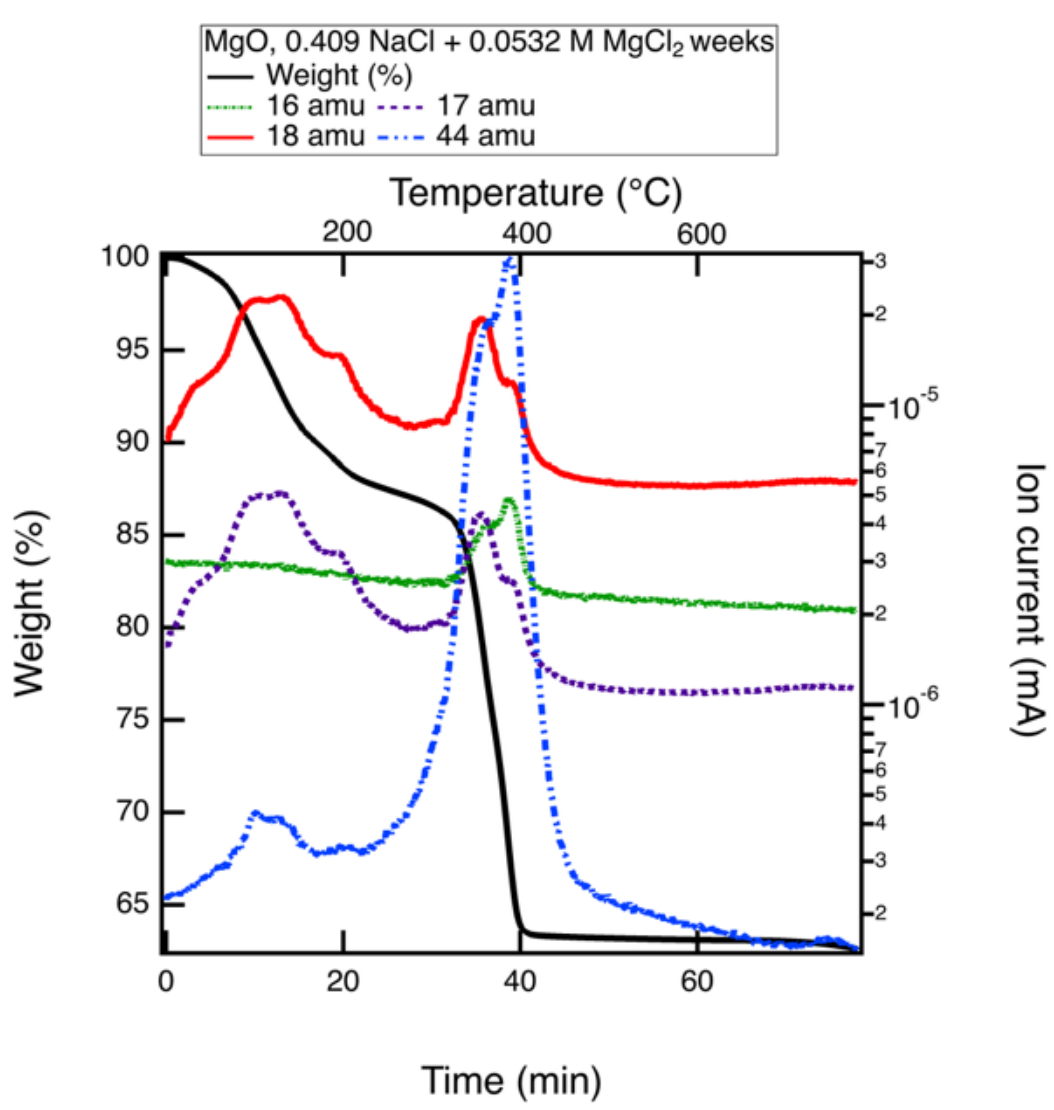


Figure S24. TGA-MS of MgO powder hydrated with 409 mM NaCl and 53.2 mM $MgCl_2$ after carbonation for 2 weeks. Heating rate 10 °C $min^{-1}$.

## 10. Surface Area Measurements for Powder Experiments

Table S 8 Surface Area Measurements of Samples from MgO Powder Hydration Experiments.

| Salt Solution used for Hydration | Surface Area ($m^2/g$) |
|---|---|
| None | 6.044 |
| 0.001 M $CaCl_2$ | 15.65 |
| 0.010 M $CaCl_2$ | 11.91 |
| 0.100 M $CaCl_2$ | 10.13 |
| 0.001M $CaCl_2$ | 12.10 |
| 0.010 M $CaCl_2$ | 7.439 |
| 0.030 M $CaCl_2$ | 9.008 |
| 0.060 M $CaCl_2$ | 12.68 |
| 0.100 M $CaCl_2$ | 4.063 |
| 0.001 M KCl | 39.49 |
| 0.010 M KCl | 20.48 |
| 0.100 M KCl | 10.59 |
| 0.001 M NaCl | 9.034 |
| 0.010 M NaCl | 8.668 |
| 0.100 M NaCl | 9.974 |
| 0.200 M NaCl | 12.85 |
| 0.300 M NaCl | 11.27 |
| 0.409 M NaCl | 12.59 |
| 0.409 M NaCl | 11.03 |
| 0.486 M NaCl | 9.644 |
| 0.409 M NaCl + $Na_2SO_4$ | 12.61 |
| 0.409 M NaCl + 0.0103 M $CaCl_2$ | 8.817 |
| 0.409 M NaCl + 0.023 M $MgCl_2$ | 11.27 |

Specific surface area has 4 significant figures based on (1) analytical balance measurements (2) amounts of $N_2$ absorbed measured by BET in instrument-generated reports.

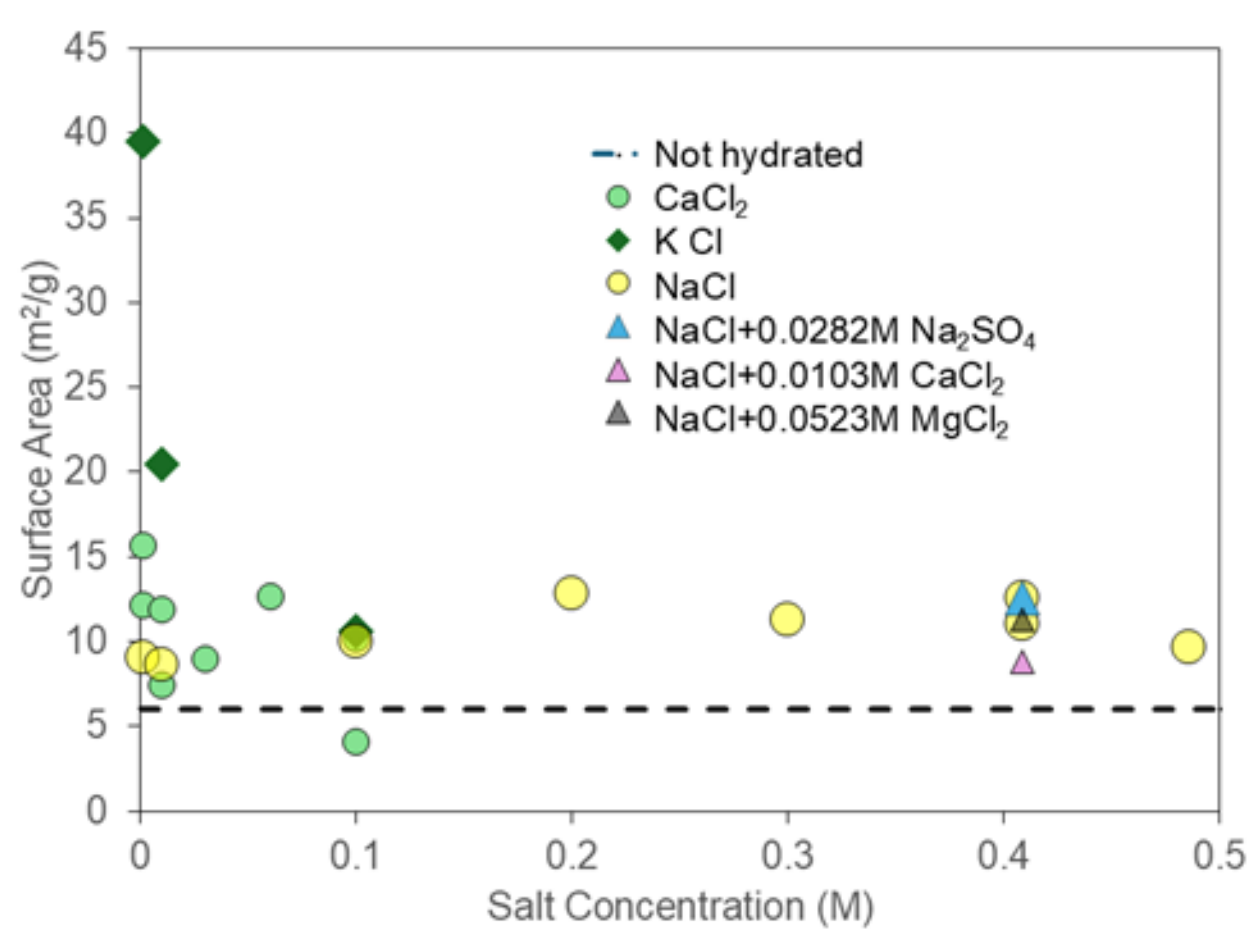


Figure S 25 Surface areas of the dried products of the 30-min hydration reactions of MgO with different salt solutions were determined by BET. Surface area of the calcined MgO before hydration was also determined (dotted line). The surface area of the calcined MgO following

hydration in purified distilled water was previously reported to be 70.3 ± 0.8 $m^2/g$ (Weber et al., 2024).

## 11. ICP-OES Analysis of MgO Hydration Supernatants

Analysis of metal content of aqueous solutions was determined with Inductively Coupled Plasma-Optical Emission Spectroscopy (ICP-OES) in radial mode using an iCAP 7000 spectrometer (Thermofisher Scientific) equipped with a prepFast M5X automatic dilution automatic sample loader (Elemental Scientific, Omaha, Nebraska). Magnesium was measured at 280.270 nm, calcium at 393.366 nm, sodium at 589.592 nm, potassium at 766 nm, lithium at 670.784 nm, and iron at 238.204 nm. Diluent and carrier solutions were 2% nitric acid (J.T. Baker Ultrapure II) in distilled water purified with a Milli-Q purification system (Millipore). Internal standard was 5 ppm yttrium. Commercial elemental standards for magnesium, sodium, calcium, and potassium (High Purity Standards, Charleston, South Carolina, USA) were used to prepare standard stock solutions containing 100 ppm Mg, 50 ppm Na, 50 ppm Ca, 50 ppm K, 50 ppm Fe, and 10 ppm Li. Automatic dilution by the sample loader was used to generate 7-point standard curves. Limit of Detection (LOD) was 0.5 ppm according to the manufacturer. Experimental solutions were filtered through syringe filters with 0.45 µm pore size. Filtrates were used to prepare 1:50 dilutions of solutions in 2% $HNO_3$. Stock solutions of the salts that were used for the experiments were similarly diluted and analyzed by ICP-OES. Standard stock solution was analyzed both as a Quality Control Check and as a sample with each run to check accuracy.

Table S9. ICP-OES Analysis of MgO single crystal reactions.

| ICP-OES 07122024 crystals | | | | Concentration [ppm] | | | | | Concentration [M] | | |
|---|---|---|---|---|---|---|---|---|---|---|---|
| Sample | reaction | Mg | Li | Na | Fe | Ca | Mg | Li | Na | Fe | Ca |
| Ca_0p001M | stock | 0.36 | -0.35 | 2.59 | 0.09 | 29.78 | 1.49E-05 | nd | 0.000112746 | 1.61155E-06 | 0.000742889 |
| Ca_0p01M | stock | 0.25 | 1.07 | 8.51 | -0.22 | 323.25 | 1.03E-05 | 0.000154733 | 0.000370252 | nd | 0.00806522 |
| Ca_0p1M | stock | 0.30 | 1.04 | 2.77 | -0.52 | 2729.67 | 1.24E-05 | 0.000149834 | 0.000120575 | nd | 0.068105464 |
| MgO_0p001MCa_1 | crystal | 1.63 | 0.30 | 5.14 | -0.08 | 28.39 | 6.69E-05 | 4.27892E-05 | 0.000223578 | nd | 0.000708209 |
| MgO_0p01MCa_1 | crystal | 3.88 | -0.48 | 2.11 | -0.20 | 313.85 | 0.00016 | nd | 9.16495E-05 | nd | 0.007830564 |
| MgO_0p1MCa_1 | crystal | 5.33 | 0.50 | 3.74 | 0.65 | 3042.96 | 0.000219 | 7.16035E-05 | 0.000162768 | 1.1621E-05 | 0.075922156 |
| MgO_0p001MCa_2 | crystal | 5.93 | 0.70 | 0.59 | -0.47 | 28.17 | 0.000244 | 0.00010013 | 2.54461E-05 | nd | 0.000702769 |
| MgO_0p01MCa_2 | crystal | 5.39 | -0.58 | -5.14 | 0.04 | 308.44 | 0.000222 | nd | nd | nd | 0.007695709 |
| MgO_0p1MCa_2 | crystal | 8.36 | -0.79 | -3.77 | -0.16 | 2955.32 | 0.000344 | nd | nd | nd | 0.073735479 |

Table S10. ICP-OES Analysis of MgO single crystal reactions.

| ICP-OES 07252024 | Concentration (ppm) | | | | | Concentration (M) | | | | |
|---|---|---|---|---|---|---|---|---|---|---|
| Sample Description | Mg | Li | Na | Fe | Ca | Mg | Li | Na | Fe | Ca |
| 0p001M_Ca_Stock_071924 | 1.00 | 1.65 | 4.10 | 0.62 | 35.13 | 0.0000412 | 0.0002373 | 0.0001781 | 0.0000111 | 0.0008766 |
| 0p01M_Ca_Stock_071924 | 0.59 | 3.25 | 3.22 | -0.11 | 343.76 | 0.0000242 | 0.0004688 | 0.0001398 | n.d. | 0.0085768 |
| 0p1M_Ca_Stock_071924 | 0.64 | 0.31 | 6.16 | 0.41 | 3214.01 | 0.0000262 | 0.0000452 | 0.0002679 | 0.0000073 | 0.0801897 |
| MgO_0p001M_Ca_5d | 2.96 | 0.39 | 4.64 | 0.08 | 34.95 | 0.0001219 | 0.0000558 | 0.0002016 | 0.0000015 | 0.0008721 |
| MgO_0p001M_Ca_3 | 10.22 | 2.20 | -0.23 | 0.17 | 34.61 | 0.0004206 | 0.0003165 | n.d. | 0.0000030 | 0.0008635 |
| MgO_0p01M_Ca_3 | 9.19 | 0.72 | 6.68 | 0.35 | 330.65 | 0.0003782 | 0.0001036 | 0.0002903 | 0.0000062 | 0.0082496 |
| MgO_0p1M_Ca_3 | 12.66 | 1.45 | 2.26 | 0.02 | 3142.33 | 0.0005207 | 0.0002083 | 0.0000984 | 0.0000003 | 0.0784013 |

Table S11. ICP-OES Analysis of MgO single crystal and powder reactions.

| ICP-OES Analysis 06-27-2024 | | Concentrations (ppm) | | | | | | Concentrations (M) | | | | | |
|---|---|---|---|---|---|---|---|---|---|---|---|---|---|
| Sample Description | Reaction | Mg | Li | Na | Fe | Ca | K | Mg | Li | Na | Fe | Ca | K |
| MgO_0p001MCa_1 | crystal | 6.80 | 2.07 | 3.27 | 2.55 | 29.61 | 2.67 | 0.00028 | 0.000298 | 0.000111 | 4.56E-05 | 0.000739 | 0.002071 |
| MgO_0p01MCa_1 | crystal | 3.92 | 1.30 | 8.45 | -0.24 | 297.65 | -7.85 | 0.000161 | 0.000187 | 0.000368 | nd | 0.007426 | nd |
| MgO_0p1MCa_1 | crystal | 5.30 | 0.16 | -0.19 | 0.26 | 2901.51 | -10.70 | 0.000218 | 2.29E-05 | nd | 4.58E-06 | 0.072393 | nd |
| CaCl2AA_1mM | stock | 0.16 | 0.31 | 1.22 | -0.31 | 41.23 | -10.73 | 6.67E-06 | 4.48E-05 | 5.32E-05 | nd | 0.001029 | nd |
| CaCl2AA_10mM | stock | 0.17 | -0.82 | 0.11 | -0.57 | 438.24 | -1.31 | 6.91E-06 | nd | 4.65E-06 | nd | 0.010934 | nd |
| CaCl2AA_100mM | stock | 0.19 | -0.86 | 2.70 | -0.06 | 4223.48 | -8.03 | 7.82E-06 | nd | 0.000118 | nd | 0.105376 | nd |
| MgO_1mMCaAA | powder | 60.61 | -0.61 | 2.41 | -0.33 | 4.18 | 176.00 | 0.002494 | nd | 0.000105 | nd | 0.000104 | 0.004501 |
| MgO_10mMCaAA | powder | 65.30 | 0.77 | 7.03 | -0.35 | 329.88 | 178.05 | 0.002686 | 0.000111 | 0.000306 | nd | 0.008231 | 0.004554 |
| MgO_100mMCaAA | powder | 83.46 | 0.48 | 11.48 | -0.17 | 4051.51 | 143.27 | 0.003434 | 6.9E-05 | 0.000499 | nd | 0.101086 | 0.003664 |
| MgO_CaACROS | powder | 86.80 | -0.53 | 20.87 | -0.09 | 4013.96 | 159.78 | 0.003571 | nd | 0.000908 | nd | 0.100149 | 0.004087 |
| SeaSim2_Stock | stock | 1317.87 | 0.06 | 9924.82 | 0.29 | 1.58 | -9.49 | 0.054222 | 7.92E-06 | 0.431706 | 5.16E-06 | 3.94E-05 | nd |
| SeaSim3_Stock | stock | 0.16 | -0.62 | 11012.05 | -0.15 | 1.37 | -7.91 | 6.58E-06 | nd | 0.478998 | nd | 3.42E-05 | nd |
| SeaSim4_Stock | stock | 0.16 | 0.00 | 10417.46 | -0.27 | 439.04 | -11.78 | 6.67E-06 | 2.88E-07 | 0.453135 | nd | 0.010954 | nd |
| MgO_SeaSim1A | powder | 60.37 | -0.28 | 10396.82 | -0.61 | 1.62 | 113.57 | 0.002484 | nd | 0.452237 | nd | 4.04E-05 | 0.002905 |
| MgO_SeaSim2 | powder | 1399.67 | -0.16 | 10336.28 | -0.28 | 1.66 | 129.54 | 0.057588 | nd | 0.449603 | nd | 4.13E-05 | 0.003313 |
| MgO_SeaSim3 | powder | 56.26 | 0.29 | 11420.16 | -0.52 | 1.53 | 149.21 | 0.002315 | 4.13E-05 | 0.49675 | nd | 3.8E-05 | 0.003816 |
| MgO_SeaSim4 | powder | 67.15 | -0.75 | 9740.89 | -0.31 | 297.84 | 122.57 | 0.002763 | nd | 0.423705 | nd | 0.007431 | 0.003135 |
| SEAWATER SIMULANT COMPOSITION | | | | | | | | | | | | | |
| SeaSim1A: 0.409M NaCl | | | | | | | | | | | | | |
| SeaSim2: 0.409M NaCl, 0.0535 M MgCl2 | | | | | | | | | | | | | |
| SeaSim3: 0.409 M NaCl, 0.0282 M Na2SO4 | | | | | | | | | | | | | |
| SeaSim4: 0.409 M NaCl, 0.0103 M CaCl2 | | | | | | | | | | | | | |

Table S12. ICP-OES Analysis of MgO powder reactions.

| ICP-OES 04232024 | Average (ppm) | | | | | Molarity (M) | | | | |
|---|---|---|---|---|---|---|---|---|---|---|
| Sample | Mg | Ca | Li | Na | Fe | Mg | Ca | Li | Na | Fe |
| stock_0p001MCaCl2 | 0.446 | 46.980 | 2.570 | 5.284 | 0.018 | 0.00001835 | 0.001172 | 0.00037 | 0.00023 | 3.22E-07 |
| MgO_0p001M CaCl2 | 24.587 | 19.091 | 7.500 | 9.967 | -0.037 | 0.0010116 | 0.000476 | 0.00108 | 0.000434 | nd |
| stock_0p01MCaCl2 | 0.190 | 449.240 | -0.578 | 2.076 | -0.069 | 7.82E-06 | 0.011208 | nd | 9.03E-05 | nd |
| MgO_0p01M CaCl2 | 29.158 | 411.846 | 5.442 | 8.334 | -0.233 | 0.001199 | 0.010275 | 0.000596 | 0.000363 | nd |
| stock_0p1MCaCl2 | 0.173 | 4292.627 | 4.856 | 8.882 | 0.060 | 7.12E-06 | 0.1071 | 0.0007 | 0.000386 | 1.07E-06 |
| MgO_0p1MCaCl2 | 39.019 | 4236.321 | 2.357 | 8.931 | 0.154 | 0.0016053 | 0.10569 | 0.00034 | 0.000388 | 2.76E-06 |

Table S13. ICP-OES Analysis of MgO powder reactions.

| ICP_OES Analysis | Concentration (ppm) | | | | | | concentration (M) | | | | | |
|---|---|---|---|---|---|---|---|---|---|---|---|---|
| 5102024 | Mg | Li | Na | Fe | Ca | K | Mg | Li | Na | Fe | Ca | K |
| NaCl 0.001M stock | 0.61 | 2.621 | 44.103 | -0.126 | 1.732 | -2.071 | 0.000025097 | 0.000378 | 0.0019183 | n.d. | 4.32E-05 | n.d. |
| NaCl 0.01M stock | 0.207 | 2.775 | 271.598 | -0.324 | 1.334 | 2.86 | 8.5167E-06 | 0.0004 | 0.011813 | n.d. | 3.33E-05 | 0.000073148 |
| NaCl 0.1M stock | 0.173 | 3.616 | 2448.771 | -0.312 | 1.397 | 6.719 | 7.12E-06 | 0.000521 | 0.10651 | n.d. | 7.31E-05 | 0.00017184 |
| NaCl 0.486 M stock | 0.133 | 2.11 | 7015.018 | -1.328 | 2.238 | 16.734 | 5.47E-06 | 0.000304 | 0.30513 | n.d. | 5.58E-05 | 0.00042799 |
| MgO_0p001M_ NaCl | 27.549 | 2.177 | 31.125 | -0.387 | 1.447 | 67.661 | 0.0011334 | 0.000314 | 0.0013538 | n.d. | 3.61E-05 | 0.0017305 |
| MgO_0p01M_NaCl | 25.317 | 1.285 | 252.271 | -0.321 | 1.811 | 57.423 | 0.0010416 | 0.000185 | 0.010973 | n.d. | 4.52E-05 | 0.0014686 |
| MgO_p0.1M_NaCl | 27.098 | 2.644 | 2365.559 | -0.313 | 1.504 | 47.078 | 0.0011149 | 0.000381 | 0.10289 | n.d. | 3.75E-05 | 0.001204 |
| MgO_0.486M_NaCl | 31.94 | 9.352 | 10742.31 | -0.651 | 1.533 | 100.517 | 0.0013141 | 0.001347 | 0.46726 | n.d. | 3.82E-05 | 0.0025708 |
| KCl 0.001M stock | 0.158 | 1.759 | 13.106 | -0.497 | 1.462 | 38.391 | 6.50E-06 | 0.000253 | 0.000076512 | n.d. | 3.65E-05 | 0.0009819 |
| KCl 0.01M stock | 0.188 | 1.619 | 12.524 | -0.243 | 1.918 | 427.697 | 0.000007735 | 0.000233 | 0.000070422 | n.d. | 4.79E-05 | 0.01093 |
| KCl 0.1M stock | 0.137 | 3.307 | 16.939 | -0.255 | 1.304 | 3884.913 | 5.64E-06 | 0.000476 | 0.0007368 | n.d. | 3.25E-05 | 0.09936 |
| MgO_0p001M_KCl | 4.809 | 0.76 | 8.536 | -0.245 | 1.314 | 83.478 | 0.00019786 | 0.000109 | 0.00037129 | n.d. | 3.28E-05 | 0.002135 |
| MgO_0p01M_KCl | 26.653 | 1.042 | 10.366 | -0.055 | 1.466 | 360.216 | 0.0010966 | 0.00015 | 0.00045089 | n.d. | 3.66E-05 | 0.009213 |
| MgO_0p1M_KCl | 28.959 | 2.294 | 16.343 | -0.528 | 1.773 | 4088.723 | 0.0011914 | 0.00033 | 0.00071088 | n.d. | 4.42E-05 | 0.10457 |

Table S14. ICP-OES Analysis of MgO powder reactions.

| ICP-OES 10042024 | Concentration (ppm) | | | | | Concentration (M) | | | | |
| --- | --- | --- | --- | --- | --- | --- | --- | --- | --- | --- |
| Sample Description | Mg | Li | Na | Fe | Ca | Mg | Li | Na | Fe | Ca |
| 0p2M_NaCl_Stock | 0.192 | 2.325 | 4807.513 | 0.097 | 0.682 | 0.0000079 | 0.0003350 | 0.20911 | 0.0000017 | 0.0000170 |
| 0p3M_NaCl_Stock | 0.135 | 2.486 | 7070.886 | 0.158 | 0.835 | 0.0000056 | 0.0003582 | 0.30756 | 0.0000028 | 0.0000208 |
| 0p409M_NaCl_Stock | 0.174 | -0.14 | 9671.957 | 0.036 | 1.687 | 0.0000072 | n.d. | 0.42070 | 0.0000006 | 0.0000421 |
| MgO_0p2M_NaCl | 55.487 | 3.019 | 4688.991 | -0.136 | 1.057 | 0.0022829 | 0.0004350 | 0.20396 | n.d. | 0.0000264 |
| MgO_0p3M_NaCl | 56.771 | -0.87 | 7278.865 | 0.045 | 1.462 | 0.0023358 | n.d. | 0.31661 | 0.0000008 | 0.0000365 |
| MgO_op409M_NaCl | 53.647 | 0.088 | 9706.274 | -0.054 | 1.151 | 0.0022072 | 0.0000127 | 0.42220 | n.d. | 0.0000287 |

## 12. Additional SEM Characterization

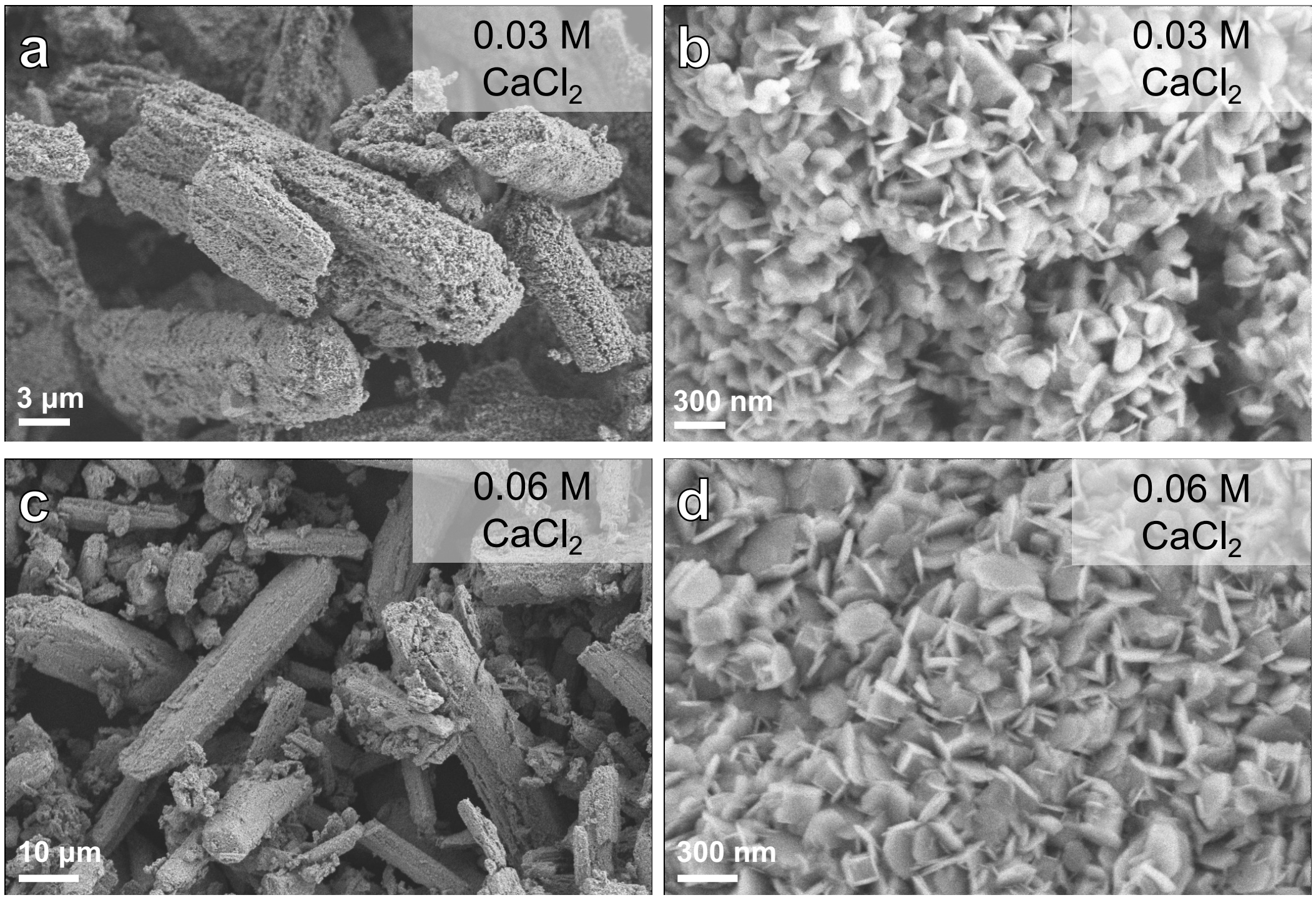


Figure S 26. Scanning electron microscopy characterization of MgO hydrated in presence of calcium chloride. a) Secondary electron (SE) contrast image of MgO hydrated in 0.03 M $CaCl_2$, b) higher magnification image of platelets formed during hydration in 0.03 M $CaCl_2$. C) SE image of MgO hydrated in 0.06 M $CaCl_2$, d) platelets formed during hydration in 0.06 M $CaCl_2$.

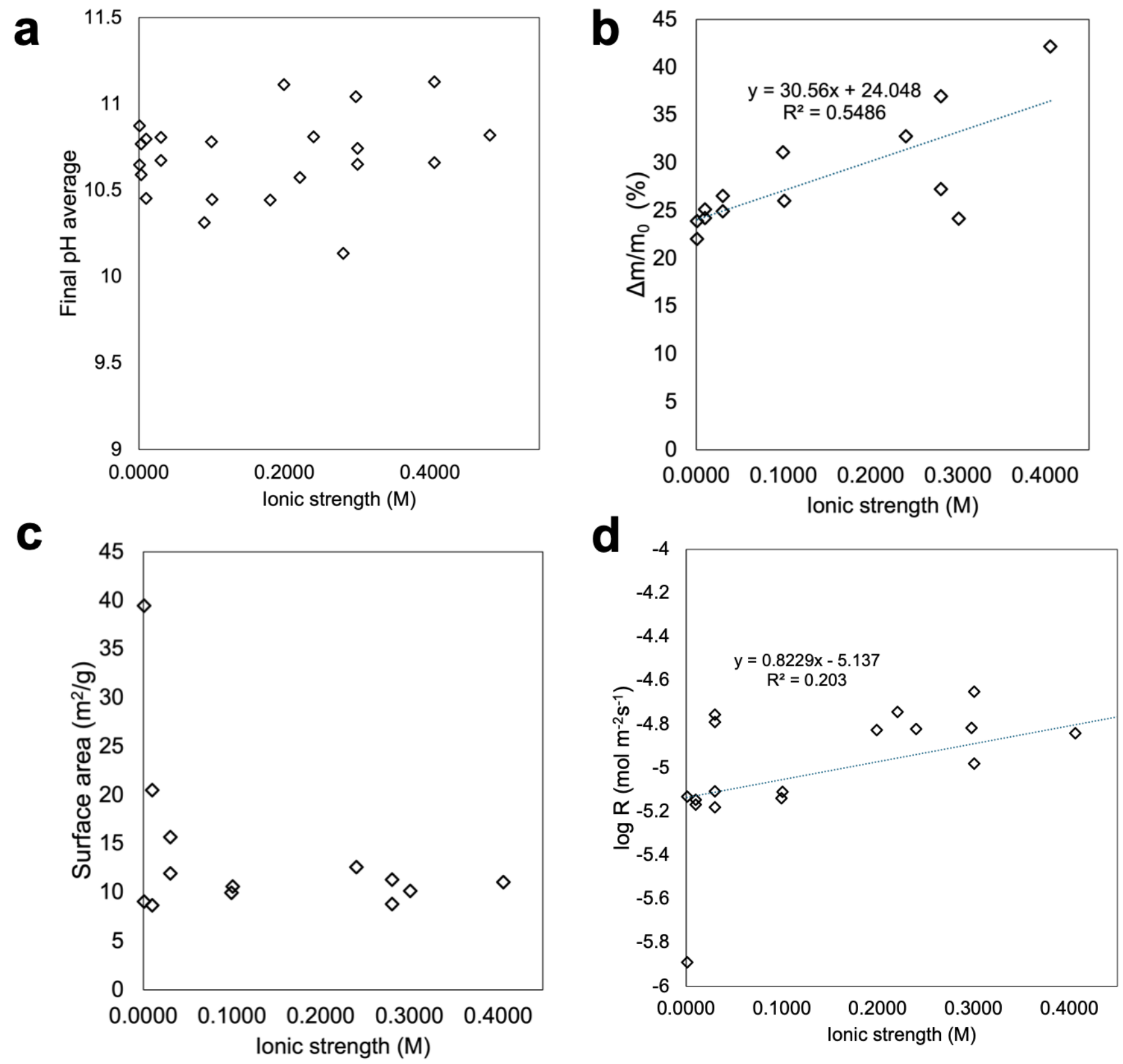


Figure S 27. Testing of effects of ionic strength. Final pH (a), mass loss during carbonation (b), surface area measured by BET (c) and logarithm of the dissolution rate (d) as a function of ionic strength. Carbonation experiments were not conducted for all hydration experiments.

## 13. References

(1) Kester, D. R.; Duedall, I. W.; Connors, D. N.; Pytkowicz, R. M. Preparation of artificial seawater. *Limnology and oceanography* **1967**, *12* (1), 176-179.

(2) Weber, J.; Moseley, B.; Yuan, K.; Evans, B. R.; Starchenko, V.; Tajuelo Rodriguez, E.; Chung, D. Y.; Boebinger, M. G.; McGuire, M. A.; Yumnam, G. Influence of Dissolved Iron in Solution on MgO Hydroxylation and Carbonation. The Journal of Physical Chemistry C 2024, 129 (1), 194-204.
